# Immunomodulatory role of metalloproteases in cancers: Current progress and future trends

**DOI:** 10.3389/fimmu.2022.1064033

**Published:** 2022-12-16

**Authors:** Qi Wang, Kai Wang, Xiaojing Tan, Zhenxiang Li, Haiyong Wang

**Affiliations:** ^1^ Department of Radiation Oncology, Shandong Cancer Hospital and Institute, Shandong First Medical University and Shandong Academy of Medical Sciences, Jinan, China; ^2^ Key Laboratory of Epigenetics and Oncology, Research Center for Preclinical Medicine, Southwest Medical University, Luzhou, China; ^3^ Department of Oncology, Dongying People's Hospital, Dongying, China; ^4^ Department of Medical Oncology, Shandong Cancer Hospital and Institute, Shandong First Medical University and Shandong Academy of Medical Sciences, Jinan, China

**Keywords:** metalloproteases, immunomodulatory, cancers, diagnosis, therapy

## Abstract

Metalloproteinases (MPs) is a large family of proteinases with metal ions in their active centers. According to the different domains metalloproteinases can be divided into a variety of subtypes mainly including Matrix Metalloproteinases (MMPs), A Disintegrin and Metalloproteases (ADAMs) and ADAMs with Thrombospondin Motifs (ADAMTS). They have various functions such as protein hydrolysis, cell adhesion and remodeling of extracellular matrix. Metalloproteinases expressed in multiple types of cancers and participate in many pathological processes involving tumor genesis and development, invasion and metastasis by regulating signal transduction and tumor microenvironment. In this review, based on the current research progress, we summarized the structure of MPs, their expression and especially immunomodulatory role and mechanisms in cancers. Additionally, a relevant and timely update of recent advances and future directions were provided for the diagnosis and immunotherapy targeting MPs in cancers.

## 1 Introduction

Metalloproteinases (MPs) efficiently hydrolyze proteins and peptides. MPs comprise the largest of the five groups of proteases in the human genome and can be divided into two subgroups: endopeptidase and exopeptidase. Endopeptidases are split into three main families; matrix metalloproteinases (MMPs), A disintegrin and metalloproteases (ADAMs), and ADAMs with thrombospondin motifs (ADAMTS) (1, 2). It is well known that metalloproteinases initially produced were in inactive zymogens form, which were activated by intracellular and extracellular proteins such as plasmin and even active members of their family (3). And recently, several new mechanisms have been found increasing a few complications of the regulation of metalloproteinases activity, including binding to the molecules of ECM or some specific cells, such as cancers cells and immune cells (4). For instance, the proteolytic activity of MMP7 can be increased after it’s catalytic site interacted with cholesterol sulfate on the surface of colon cancer cells (5). Active metalloproteinases are dependent on metal ions that are inhibited by tissue inhibitors of metalloproteases (TIMPs) as well as other metal chelating agents such as ethylene diamine tetra-acetic acid and phenanthroline (2, 6, 7).

Studies have shown that MPs are involved in multiple biological and pathological processes including inflammatory and immune interactions, protein homeostasis, processing of peptide hormones, release of cytokines and growth factors, metabolism of antibiotics, cell migration and invasion and tissue morphogenesis (8, 9). Recent studies have demonstrated that MPs and their inhibitors are closely related to the diagnosis, treatment and prognosis of cancers, especially in immunomodulation during tumorigenesis and cancer development (10–12). In fact, MPs not only regulate tumor immunomodulation through signal transduction pathways, but also influence the tumor microenvironment (TME) (13, 14). Most cancers are associated with a TME rich in various immune-infiltrating cells and factors produced by these cells that induce host cells to differentiate and produce growth factors, cytokines, and chemokines that are conducive to tumor cell survival and metastasis (15). MPs can modulate immunoregulatory factors and other immune-related proteins containing cytokine receptors, notch receptors, phagocytic receptors and cell adhesion molecules, all of which play significant roles in immune system function (11, 16).

MPs are also responsible for matrix protein degradation and remodeling of the extracellular matrix (ECM), either directly or through the liberation of growth factors and cell surface receptors (14). The ECM, a non-cellular three-dimensional macromolecular network structure secreted by cells, is composed of collagen, proteoglycan/glycosaminoglycan, elastin, fibronectin, laminin and other glycoproteins (17). Proteoglycan and hyaluronic acid, which are rich in the ECM, have been found to be elevated during inflammatory progression in a variety of diseases, including cancers (18, 19). The ECM becomes highly dysregulated in tumors and the loss of matrix homeostasis is considered a hallmark of solid tumors (20). Moreover, the ECM contains bioactive motifs that can directly modulate immune responses in cancer (21). For a long time, studies of tumor invasion and metastasis have focused on the inherent adhesion and migration ability of tumor cells themselves. MPs are known to assist tumor cells to break through the tissue barrier *via* adhesion and protease hydrolysis, and facilitate ECM remodeling during the migration process, leading to invasion and metastasis (22, 23).

In this review, we provide an overview of MPs structure, expression and regulation in various tumors, principally MMPs, ADAMs and ADAMTS, with a focus on their emerging immunomodulatory role in cancers. We also review investigational MP-targeting therapies.

## 2 The structure, expression and role of MPs in cancer

**Figure 1:**
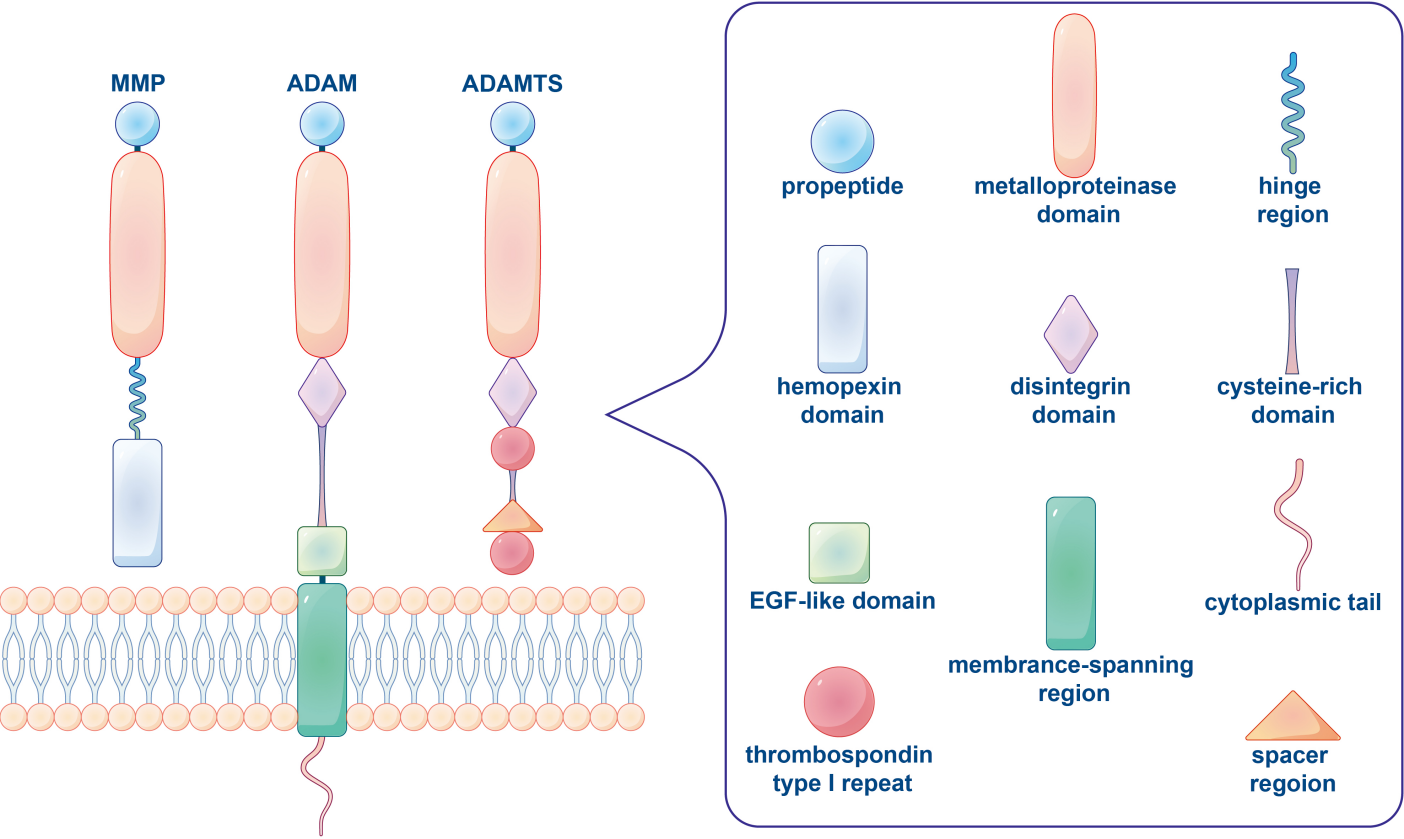
The typical domain structure of human metalloproteinases (MMP, ADAM, ADAMTS). An archetypal MMP orderly contains a propeptide, a catalytic metalloproteinase domain, a linker peptide (hinge region) and a hemopexin domain. ADAM is a class of transmembrane protein with characterized structure including a propeptide, a metalloproteinase domain, a disintegrin domain, a cysteine-rich domain, an EGF-like domain, a membrane-spanning region and a cytoplasmic tail. The basic domain organization of ADAMTS comprises a propeptide, a metalloproteinase and disintegrin-like domain, a thrombospondin type 1 repeat (TSR), a cysteine-rich domain and a spacer region, while lacked a transmembrane region, cytoplasmic domain and EGF-like domain.

There are numerous members of the MP family that have similar but not identical structure. Importantly, each MP has a varying but equally important roles in the occurrence and development of various cancers (**Figure 1** and **Table 1**).

**Table 1:** The prognostic role and functions of several representative metalloproteinases involved in pan-cancers.

Type of MP	Representatives	Cancers	Prognostic role	Functions	References
Matrix Metalloproteinase (MMPs)	MMP2	Most types of cancer	Decreasing OS	Degrading collagen and extracellular matrix; Involving TGF-β signaling pathway; Increasing cytokine production; Enhancing cancer cell invasiveness; Promoting angiogenesis;	(24–37)
	MMP9	Most types of cancer	Decreasing OS	Degrading collagen and extracellular matrix; Involving TGF-β signaling pathway; Facilitating the migration of immune cell; Hydrolyzing cytokines; Promoting cancer migration, invasiveness and metastasis; Promoting angiogenesis;	(24–29, 31–35, 38–50)
	MMP14	Ovarian cancer, Breast cancer, etc.	Decreasing OS	Degrading extracellular matrix; Cleaving and activating cytokines; Activating metalloproteinase; Promoting angiogenesis;	(27–29, 31–35, 39, 51)
A Disintegrin and Metalloprotease (ADAMs)	ADAM10	Lung cancer, glioma, head and neck squamous cell carcinoma(HNSCC), etc.	Most in decreasing OS (controversy in HNSCC)	Involving Notch signaling pathway; Involving formation of cancer-associated fibroblasts; Regulating immune cells; Inhibiting antitumor immunity;	(13, 52–69)
	ADAM17	Most types of cancer	Decreasing OS	Involving TNF and Notch signaling pathway; Regulating immune cells; Regulating cytokines; Inhibiting antitumor immunity;	(12, 13, 53, 54, 58–63, 67, 68, 70–78)
ADAMs with Thrombospondin Motifs (ADAMTS)	None	Many cancers	Uncertainty	Dual function (Promoting and inhibiting cancer progression)	(79–82)

### 2.1 Matrix metalloproteinases

The MMP family consists of 28 members, at least 23 of which are expressed in human tissues (83). Typically MMPs comprise a pro-peptide of about 80 amino acids maintaining the stability of zymogen, a catalytic metalloproteinase domain of about 170 amino acids with zinc ion binding sites, a linker peptide (hinge region) of variable length rich in proline, and a hemopexin domain of about 200 amino acids in length (84, 85). Although MMPs have a common core structure, they are classified either as collagenases (MMP1, MMP8, MMP13), gelatinases (MMP2, MMP9), stromelysins (MMP3, MMP10, MMP11), matrilysins (MMP7, MMP26), membrane-type MMPs (MT-MMPs) or other MMPs according to the structure of their substrates and structural domains (86). PRCGXPD is a cysteine-switch motif in the MMP pro-peptide that is responsible for the characteristic proteolytic activity (84). Different types of MMPs have specific structural characteristics that are different from typical MMPs (86). For instance, MT-MMPs lack the pro-domain, while MMP7 (matrilysin 1), MMP26 (matrilysin 2) and MMP23 lack the Hpx domain and the linker peptide. In addition, MMP2 and MMP9 contain three repeats of a fibronectin. These various domains, modules, and motifs in MMPs are involved in interactions with other molecules, thereby influencing or determining MMP activity, substrate specificity, cellular and tissue localization (87). MMPs participate in both broad-spectrum turnover and some proteolysis of extracellular proteins, which includes ectodomain shedding at the plasma membrane in a complementary fashion to ADAMs (24). In addition, MMPs can degrade the ECM and participate in almost the whole process of tumor growth and development, including tumor invasion, metastasis and angiogenesis (38).

Reportedly, MMPs have been detected in a variety of human cancers, and high expression of MMPs is usually associated with poor survival in most cancers (25) including colorectal cancer (39), lung cancer (40), breast cancer (26), ovarian cancer (41), and gastric cancer (70). However, upregulation of a specific MMP does not always lead to promotion of tumor growth or metastasis. The gelatinase protein family including MMP2 (gelatinase A) and MMP9 (gelatinase B) are able to degrade type IV collagen in basement membranes and are the most extensively studied metalloproteinases that are associated with disease progression and poorer survival in patients with various cancers (88). A meta-analysis comprising 41 studies and 6517 patients with primary breast cancer reported that MMP2 and MMP9 overexpression conferred a higher risk of distant and lymph node metastasis, respectively, and were both associated with higher clinical stage and histological grade (26). Similarly, high levels of MMP12 are strongly associated with poor survival in patients with gastric cancer. In contrast, MMP12 overexpression is related to increased survival in patients with colorectal cancer (70, 89).

### 2.2 A disintegrin and metalloproteases

Also known as Metalloprotease Disintegrin Cysteine-rich (MDC), ADAMs are type I transmembrane proteins anchored to cell surface membranes. Over 30 types of ADAM have been discovered so far (90). Similar to MMPs, ADAMs include a pro-domain and a zinc-binding metalloprotease domain. ADAMs also include a disintegrin domain, which is unique among cell surface proteins (91). The metalloproteinase domain of ADAM is highly conserved, and most ADAMs have an EGF-like domain adjacent to a cysteine-rich domain and a membrane-spanning region, followed by a cytoplasmic tail that varies widely in length and sequence between different ADAM family members (90–92). Due to the presence of these domains, ADAMs can bind substrates and affect changes in cell adhesion and migration, and the proteolytic release of cell surface molecules. Their major substrates are intact transmembrane proteins such as growth factors, adhesion molecules, and precursor forms of cytokines (71).

Cancer cells often express high levels of ADAMs, suggesting a selective advantage for tumors. Interestingly, overexpression of ADAMs is not carcinogenic (52). There are two functional attributes of ADAM proteins, namely proteolytic activity and cell adhesion, which supports the hypothesis that ADAMs may have a crucial role in cell migration as well as extracellular remodeling (93) ADAM17 is the most widely studied of all the ADAM proteins. One study evaluating ADAM17 as a potential blood biomarker for ovarian cancer showed that ADAM17 levels are significantly higher in culture medium supernatants of cultured ovarian cancer cell lines and also in the serum and ascites of patients with ovarian cancer, compared with controls (94). Toshie et al. reported that ADAM10 could be a potential lung cancer biomarker through investigating enzyme-specific proteolytic activities, rather than ADAM17 (95). Proteomics technologies can be used to identify ADAM proteins that are shed by tumor cells (52).

### 2.3 ADAMs with thrombospondin motifs

As close relatives of ADAMs, ADAMTS also belong to the Adamalysins family of proteins (79, 80). Unlike ADAMs, ADAMTS are secreted metalloproteinases characterized by an ancillary domain containing a thrombospondin type 1 repeat (TSR) and a spacer region, and the absence of a transmembrane region, cytoplasmic domain and (EGF)-like (81). The human ADAMTS family includes 19 proteins that can be sub-grouped on the basis of their known substrates, namely aggrecanases or proteoglycanases (ADAMTS1, 4, 5, 8, 9, 15 and 20), procollagen N-propeptidases (ADAMTS2, 3 and 14), cartilage oligomeric matrix protein (also known as thrombospondin-5), cleaving proteinases (ADAMTS7 and 12), von Willebrand factor (VWF), cleaving proteinase (ADAMTS13) and a group of orphan enzymes (ADAMTS6, 10, 16, 17, 18 and 19) (81, 82). ADAMTS proteases are involved in the maturation of procollagen and von Willebrand factor, as well as in ECM proteolysis relating to morphogenesis, angiogenesis and cancer (82, 96, 97).

Although major ADAMTS members were identified as suppressors or oncogenes in cancers, studies have shown that different ADAMTS exhibit diverse biological functions and that individual ADAMTS can play distinct roles in different cancers or depending on the clinical setting (98). For example, high expression of ADAMTS8 was associated with better survival in patients with lung cancer by inhibiting cell proliferation and promoting apoptosis.ADAMTS8 overexpression was also associated with decreased levels of vascular endothelial growth factor A (VEGFA), which is a major regulator of angiogenesis and contributes to tumor growth and metastasis (99). A study using topological data analysis identified 38 elusive cancer-related genes, including an inactivating mutation in ADAMTS12 in lung adenocarcinoma. Mice with ADAMTS12 deletion mutations have a five-fold increased susceptibility to lung cancer, confirming the role of ADAMTS12 as a tumor suppressor gene (100). In general, the involvement of ADAMTS in the TME is less well studied compared with MMPs and ADAMs, and studies systematically characterizing their functions in cancers are urgently needed.

## 3 The relationship between metalloproteases and immunomodulation in cancers

### 3.1 Signal pathway involving MPs related to immunity in cancer cells

**Figure 2:**
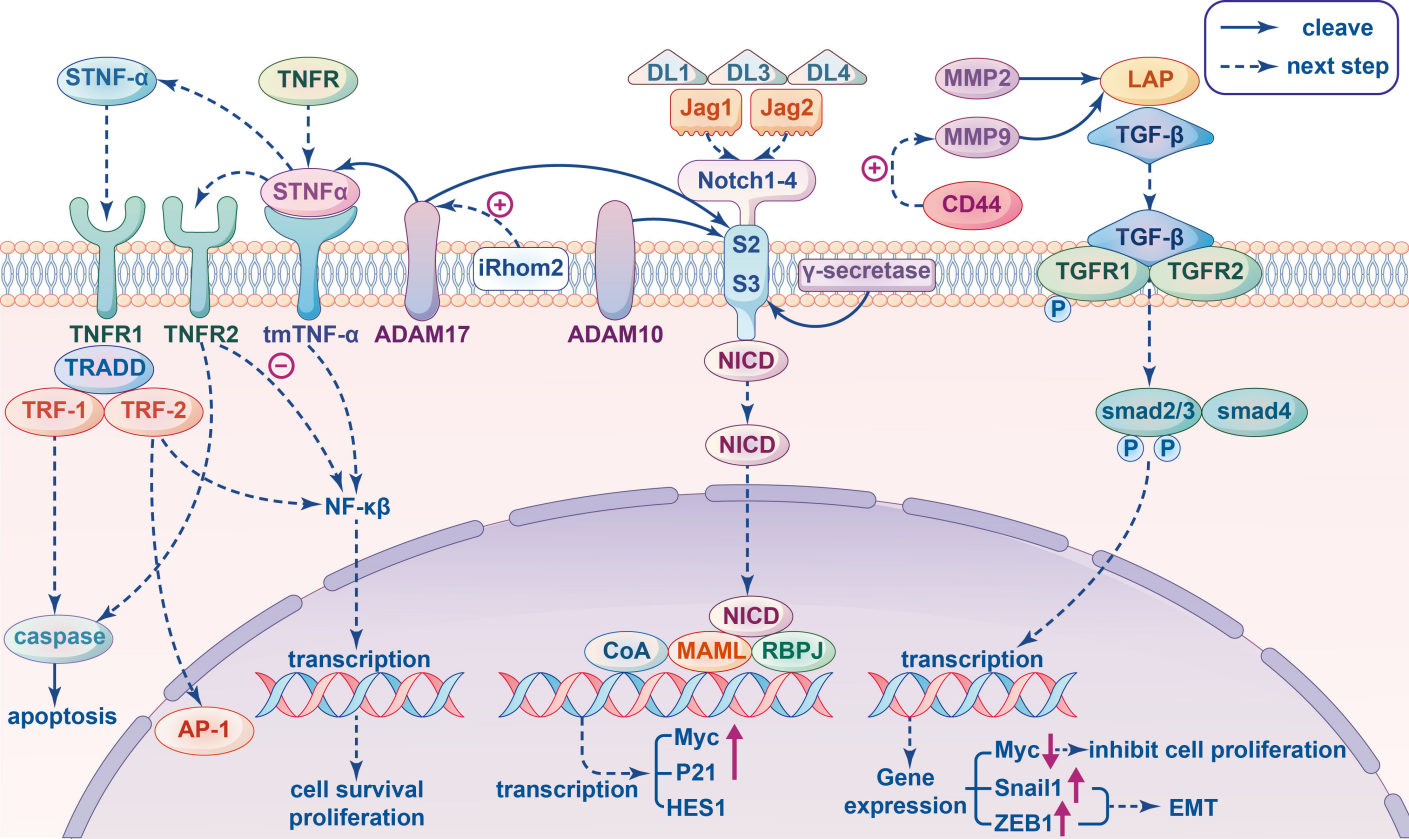
Signal pathway involving metalloproteinases related to immunity in cancer cells. Tumor Necrosis Factor (TNF) signal pathway: The transmembrane TNF-α (tmTNF-α) cleaved by proteolytic enzyme ADAM17 produced soluble TNF-α (sTNF-α), which binds to TNFR1 and then recruits TNFR-associated death domain (TRADD), TNFR-associated factor (TRF)-1 and TRF-2 generating two different results: mediating caspase activation to apoptosis or leading to activation of the NF-κB and AP-1 for tumor cell proliferation. When tmTNF-α binds to TNFR2 as ligands to inhibite NF-κB mediated activation of anti-apoptotic genes, then apoptosis and tumor suppression will be triggered; as a receptor tmTNF-α transect the reverse signal to promote tumor proliferation by constitutively activating NF-κB. The activity of ADAM17 can be stimulate by pseudoprotease enzyme iRhom2. Transforming growth factor β (TGF-β) signal pathway: MMP9 and MMP2 cleave the inactive latent TGF-β propeptide (LAP) and produce different activated TGF-β proteolytic cleavage products. In the canonical pathway, active TGF-β triggers TGFβRII to phosphorylate TGFβRI, which in turn recognizes and phosphorylate SMAD2 and SMAD3 proteins to interact with SMAD4 to form a complex that can enter the nucleus and regulate the transcription of target genes. For example, down-regulating the expression of proto-oncogene Myc to inhibit cell proliferation, inducing the Snail1 and ZEB1 to promote epithelial mesenchymal transition (EMT) in tumors. The co-aggregation of CD44 and MMP9 can promoted the protein activity of MMP9. Notch signaling pathway: ADAM10 and ADAM17 have been indicated in many studies that they can act on cleaving S2, and subsequent cleavage S3 mediated by γ-secretase occurs in the transmembrane region, leading to the release of Notch intracellular domain (NICD), which translocate into the nucleus and combines Mastermind-like (MAML) and DNA-binding protein Recombination Signal-binding Protein for Immunoglobulin kappa J region (RBPJ) to recruits additional coactivators (CoA) triggering the transcription of target genes, such as Myc, P21, HES1 and so on.

Signal transduction pathways are comprised of multiple molecules recognizing and interacting with each other and transmitting signals to regulate many important biological processes such as tumor cell proliferation, metastasis and immune regulation. Three signaling pathways in particular are closely related to MPs in immunomodulation and are described below (**Figure 2**).

#### 3.1.1 Tumor necrosis factor signaling

Tumor necrosis factor-α (TNF-α) is an important proinflammatory cytokine that is involved in the maintenance and homeostasis of the immune system, as well as inflammation and host defense (72). Substantial experimental and clinical data have been shown that TNF-α is involved in the promotion and progression of cancer (72, 101–104). TNF-α is found in both a soluble and transmembrane form. Soluble TNF-α is cleaved from transmembrane TNF-α (tmTNF-α) by proteolytic enzyme ADAM17, also known as TNF-a-converting enzyme (TACE), which can orchestrate immune and inflammatory responses *via* activation of TNF-α (73, 90). Both tmTNF-α and soluble TNF-α initiate signaling cascades by binding to TNF-α receptors. There are two types of TNF-receptors: TNF receptor 1 (TNFR1), which is activated by soluble ligands, and TNF receptor 2 (TNFR2) that binds primarily to tmTNF-α. The shedding of tmTNF-α and TNFR both require pseudoprotease enzyme iRhom2, which is an important cofactor of ADAM17 and can stimulate the activity of ADAM17 on the cell surface to control the specificity of ADAM17 protein breakdown (105).

Soluble TNF-α and tmTNF-α all possess dual abilities to promote tumor growth and survival, while tmTNF-α has much broader anti-proliferative capabilities (106). The binding of soluble TNF-α to TNFR1 generates two different out comes. First, TNFR1 recruits TNFR-associated death domain (TRADD), TNFR-associated factor (TRF)-1 and TRF-2. If the complex activates nuclear factor κB (NF-κB), tumor cell proliferation is observed. Otherwise, the complex enters the cytoplasm and the recruitment of FAS-associated *via* death domain (FADD) mediates caspase8 activation leading to apoptosis, which was found to contribute to ADAM17-mediated shedding of TNFR1 (74, 107). It should also be noted that the activity of ADAM17 plays a crucial role in TNFR1-dependent tumor cell–induced endothelial cell death. This is because ADAM17-mediated ectodomain shedding and subsequent γ-secretase-mediated regulated intramembrane proteolysis (RIP) of TNFR1 is a prerequisite for TNF-induced cell death (108). TmTNF-α can be transduced bidirectionally as a ligand or membrane receptor. Apoptosis and tumor suppression are triggered when TmTNF-α binds to TNFR2, leading to the inhibition of NF-κB-mediated activation of anti-apoptotic genes, which is regulated in part by the actin cytoskeleton. However, if tmTNF-α is expressed on the cell surface, it will act as a receptor and transect the reverse signal to promote tumor proliferation by constitutively activating NF-κB (106). Although the exact mechanism has not been elucidated, TNF-signaling in cancer cells has an overall pro-tumor effect, promoting survival, proliferation, and evasion of immune surveillance (109, 110). Therefore, in view of the action of ADAM17 on the receptors and ligands of TNF-signaling pathway, ADAM17 is deemed to affect TNF-α signaling in a variety of ways. For example, a decrease in the production of soluble TNF-α would lead to accumulation of tmTNF-α, which would bind to TNFR2 and lead to a different biological outcome (111).

#### 3.1.2 Transforming growth factor–β signaling

As a key regulator of tumor behavior, transforming growth factor- β (TGF-β) plays an important role in tumor invasion and metastasis, immune regulation and therapeutic resistance (42). TGF-β is also the core of immune suppression in the TME, which has bidirectional effects on the immune system depending on the specific situation (43). In general, TGF‐β inhibits cell proliferation during the early stages of cancer development. Inactivation of the TGF‐β pathway or decoupling from tumor suppressor effects can promote tumor progression, which affects ECM and cell adhesion molecules, promotes metastasis and angiogenesis, and induces immune suppression. Through a classic membrane-to-nucleus signaling process, the TGF-β pathway involves direct receptor-mediated activation of small mother against decapentaplegic (SMAD) transcription factors (43). TGF-β1, TGF-β2, and TGF-β3 are three closely related isoforms secreted by an inactive complex that covalently combine mature TGF-β with the latent TGF-β pro-peptide (LAP) *via* disulfide bonds in the endoplasmic reticulum (112). There are also three known TGF-β receptors (TGFβRI, TGFβRII and TGFβRIII). In the canonical pathway, active TGF‐β triggers TGFβRII to phosphorylate TGFβRI, which in turn recognizes and phosphorylates SMAD2 and SMAD3 proteins to interact with SMAD4 to form a complex that can enter the nucleus and regulate the transcription of target genes (113).

MMP9 and MMP2 are two metalloproteinases known to cleave the inactive latent TGF‐β and produce different TGF-β proteolytic cleavage products, which leads to transforming growth factor-B activation (114). Hyaluronic acid-mediated CD44 cross-linking induced co-aggregation of CD44 and MMP9 promotes the protein degradation activity of MMP9. Moreover, degradation of fibronectin by MMP9 bound to CD44 results in release of the active TGF‐β (115). The levels of MMP9 in cancer cells may not only influence the proteolysis of TGF‐β, but also the expression of TGF‐β and substances downstream of the TGF signaling pathway. A study on the relationship between MMP9 and the TGF signaling pathway in breast cancer showed that overexpression of MMP9 in breast cancer cells not only significantly up-regulated the expression of SMAD2, SMAD3 and SMAD4, but also enhanced the phosphorylation of SMAD2 (116). Subsequently, the target gene KLF10 binds to the promoters of SMAD2 and TGF-β1 and to form a positive feedback loop regulating the TGF-β signaling pathway by inducing SMAD2 expression (117). In addition, decorin, which is expressed in the stroma of various cancers and can be cleaved by MMP2, 3, 7 and MT1-MMP, recognizes and binds to all isoforms of TGF-β to form an inactive complex, which inhibits TGF-β signaling *in vitro* and indirectly attenuates downstream signaling pathways (118).

#### 3.1.3 Notch signaling pathway

Notch signaling is involved in multiple aspects of tumor biology, and its role in the development and regulation of immune responses is complex, including shaping the immune system and components of the TME, such as intricate crosstalk between antigen presenting cells, T-cell subsets and cancer cells (119). In particular, Notch plays a crucial role in the development and maintenance of different immune cells such as adaptive T-cells, Natural killer cells and innate immune myeloid cells e.g., granulocytes, macrophages, and dendritic cells (53). Several studies have found that Notch is a target of tumor-mediated immune suppression, and reactivation of Notch in T cells may protect T cells from tumor-mediated immune suppression and enhance their anti-tumor activity (54, 119). The Notch signaling pathway mediates the activation effect after two cells come into contact with each other. Notch receptors comprise four isoforms (Notch1–4), which are single-pass transmembrane proteins that receive signals from transmembrane ligands comprised of three delta-like ligands (DLL1, DLL3, and DLL4) and two jagged ligands (Jag1 and Jag2) expressed on neighboring cells (75). Following binding of transmembrane ligands to Notch receptors, downstream signaling is mediated by some proteases including members of the ADAM family (55).

Firstly, the receptor/ligand interaction exposes the proteolytic cleavage site, S2, which is cleaved by ADAM metalloproteases. Subsequent cleavage at S3, mediated by γ-secretase occurs in the transmembrane region, leading to the release of Notch intracellular domain (NICD), which translocated into the nucleus and combines Mastermind-like (MAML) with DNA-binding protein Recombination Signal-binding Protein for Immunoglobulin kappa J region (RBPJ) to recruit additional coactivators (CoA), triggering the transcription of target genes such as Myc, P21, and HES1 (120). ADAM10 and ADAM17 are known to be involved in cleaving S2, while ADAM17 leads to ligand-independent Notch activation, and ADAM10 causes ligand-dependent activation (27, 121, 122). One study tested whether restoring Notch signaling in ADAM10-deficient mice would block tumor development and showed that the loss of ADAM10 promotes head and neck squamous cell carcinoma (HNSCC) tumorigenesis by impairing Notch signaling (28).

### 3.2 Tumor microenvironment regulation by MPs

The TME refers to the surrounding microenvironment of tumor cells including blood vessels, immune cells, fibroblasts, bone marrow-derived inflammatory cells, various signaling molecules and the ECM. Previous studies have shown that tumors can modulate their microenvironment, and in turn, the TME can influence tumor growth and spread. The TME plays a key role in regulating the immune response in cancers. Tumor cells and their microenvironment typically produce multiple immunomodulatory molecules that have either negative or positive effects on immune cell function. Thus, the TME is able to switch the immune response from tumor-destructive mode to tumor-promoting mode depending on the composition of the TME.

#### 3.2.1 The influence of MPs on the ECM

**Figure 3:**
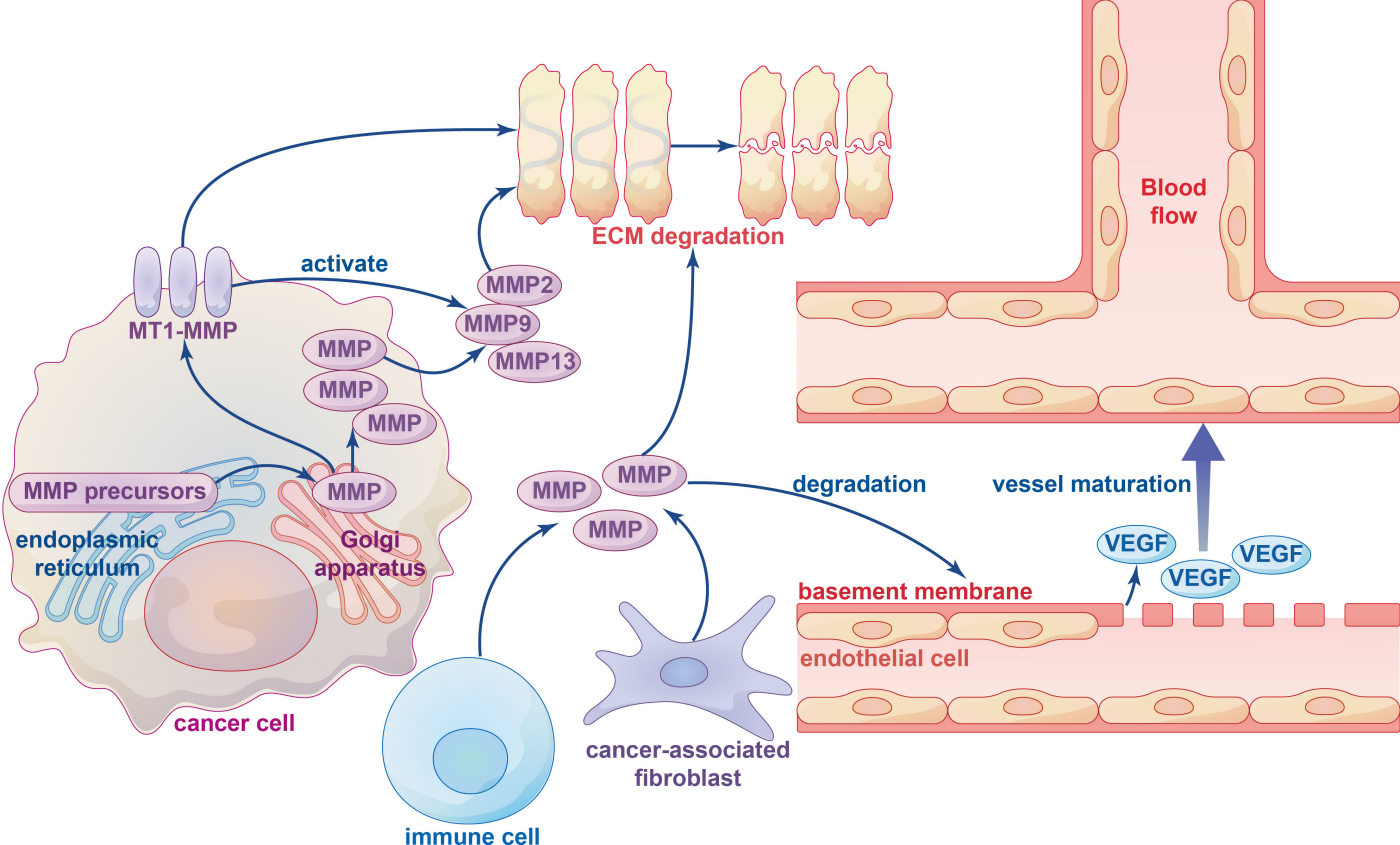
The influence of metalloproteinases on ECM degradation and angiogenesis. Various types of metalloproteinases in ECM-degrading are firstly synthesized as inactive precursors (zymogens) in the endoplasmic reticulum and then transported to the Golgi apparatus, which can be divided into membrane-type 1 matrix metalloproteinase (MT1-MMP) and other soluble MMPs. Several immune cells and cancer-associated fibroblasts (CAFs) can also produce metalloproteinases. ECM degradation is mainly performed by MT1-MMP-activated soluble MMPs, such as soluble gelatinases MMP2, MMP9 and soluble collagenase MMP13. The MMPs degrade the basement membrane structure of vascular endothelial cells and release VEGF bound to the extracellular matrix to initiate vessel maturation.

The ECM is a non-cellular component of the TME stroma, and remodeling of the ECM plays a significant role in the development and homeostasis of cancers, as well as immune cell recruitment and tissue transfer. Extensive remodeling of the ECM during cancer progression leads to changes in its density and composition, and ECM degradation is an important consequence (14). Specifically, protease-induced breakdown of ECM components is essential for tumor cells to cross tissue barriers. MMPs and ADAMs are the main enzymes involved in ECM degradation, either directly or through the release of growth factors and cell-surface receptors (29, 123). The MMPs involved in ECM degradation can be broadly divided into membrane-anchored MMPs and soluble MMPs (**Figure 3**). They are first synthesized as inactive precursors (zymogens) in the endoplasmic reticulum and then transported to the Golgi apparatus, where they are sorted and transported to specific membrane domains on the cell surface (56). Although membrane-anchored MMP14, which also called membrane-type 1 matrix metalloproteinase (MT1-MMP), localizes preferentially at membrane protrusions called invadopodia where it plays a central role in degradation of the surrounding ECM. ECM degradation is mainly achieved by MT1-MMP-activated soluble MMPs such as soluble gelatinases MMP2, MMP9 and soluble collagenase MMP13, and there is a significant decrease in total ECM degradation when soluble MMP dynamics are switched off (124–126).

The ECM has three main components: fiber, proteoglycans and polysaccharides. MMPs play an important role in tissue remodeling by binding to these substrates to promote turnover of various ECM proteins. The catalytic activity of metalloproteinases usually requires zinc ions and water molecules, and water bound to zinc ions performs a nucleophilic attack on the substrate, causing it to rupture and release the water molecules (86). Matrix degradation can also remove physical barriers (such as basement membranes), and destruction of the normal matrix facilitates malignancy and metastatic dissemination. The specific mechanism by which MMPs degrade the ECM remains unclear, although multiple studies have identified a role for MMPs in ECM degradation (125).

Cancer-associated fibroblasts (CAFs) are the main contributors to ECM stiffness and degradation, and alterations in CAFs contribute to tumor growth and dissemination as well as regulation of T-cell infiltration in cancers (14). High expression of TGF-β induces the transition of endothelial cells into mesenchymal cells, leading to the formation of CAFs and promoting tumor formation (127). In addition, a wide range of MPs controlled by the TIMP gene family influence the TME in cancer. Loss of TIMP1-4 in fibroblasts results in the acquisition of CAF-like features, manifested by increased collagen contractility and expression of activation markers such as A-SMA, stromal derived factor 1 (SDF-1), and TGF-β. ADAM10 inhibits RhoA and Notch activation induced by exonucleosome treatment, and down-regulation of ADAM10 expression in TIMP-free fibroblasts reduces their tumor-promoting and metastatic potential *in vivo (* 128). Immune cells in the TME also release MMPs to assist with ECM degradation. For instance, mast cell precursors may spontaneously produce MMP9 during local tissue migration, which is directly or indirectly activated by MMP3 released from fibroblasts, chymase released from mast cells, and plasminogen activator released from microvascular endothelial cells, thereby causing degradation of the ECM (129).

#### 3.2.2 The relationship between MPs and immune cells

**Figure 4:**
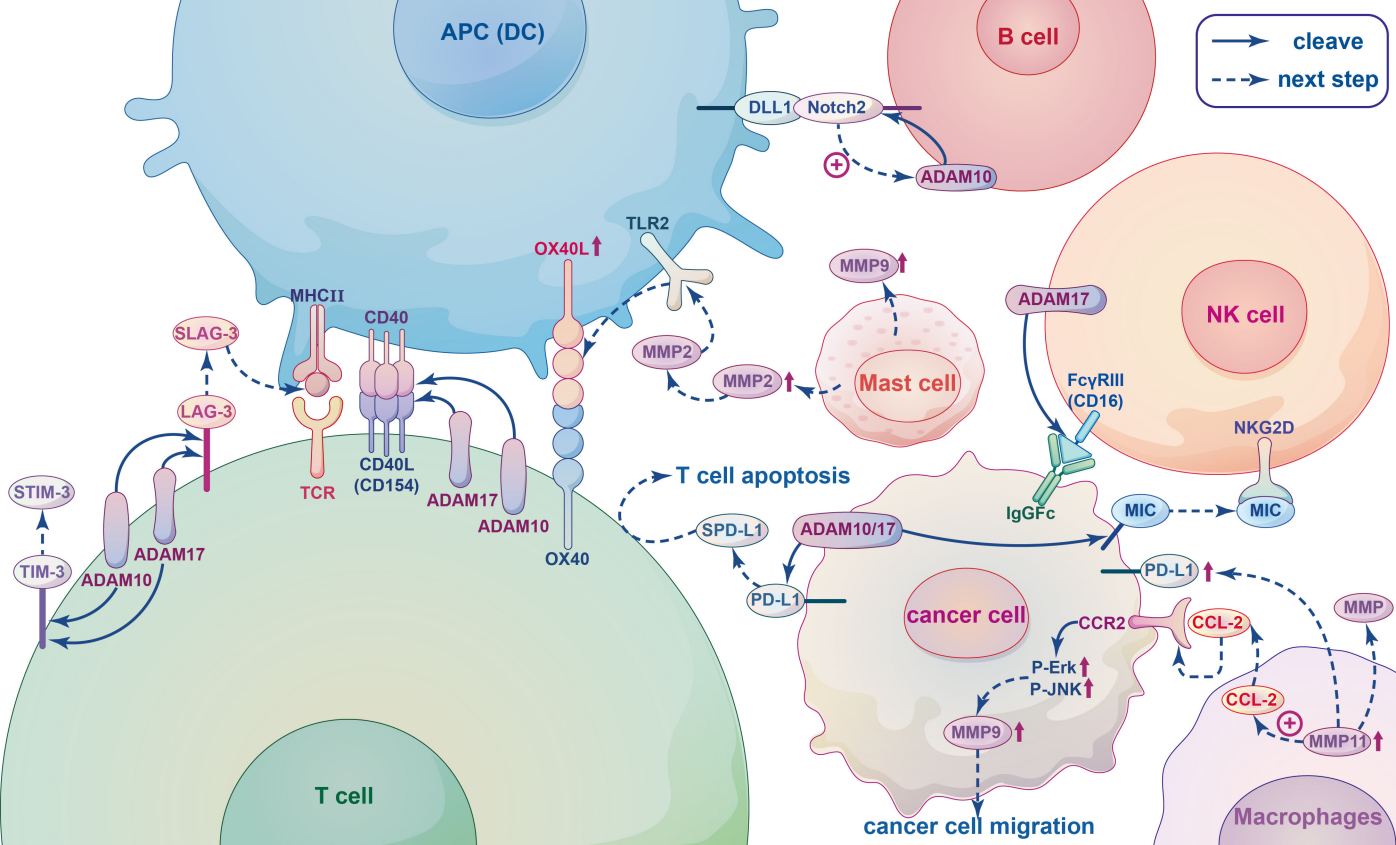
The relationship between metalloproteinases and immune cells. When T-cell receptor (TCR) of T cells interact with the major histocompatibility complex (MHC II) of antigen presenting cells (APCs), co-stimulatory receptor CD40L(CD154) expression is rapidly upregulated and linked to CD40, and subsequently released from the T cell surface by cleavage by ADAM10 and ADAM17. Lymphocyte Activation Gene-3 (LAG-3), T-cell immunoglobulin and mucin domain-containing protein-3 (TIM-3) are T-cell coinhibitory receptors can be cleaved by ADAM10 and ADAM17 yielding soluble form sLAG-3 and sTIM-3. SLAG-3 binds to MHCII inhibiting the activation of T cells and inducing dendritic cell maturation, sTIM-3 can impair the antitumor immune response of T cells. ADAM10 and ADAM17 can both produce different cleavage products of PD-L1, which called soluble PD-L1 (sPD-L1) and shed from the surface of tumor cells, inducing apoptosis of CD8+ T cells and inhibiting antitumor immunity. In B cells, Notch2 heterodimers bind to ligands DLL1 presented on antigen presenting cells (APCs), which initiates ADAM10 resulting in the release of the Notch intracellular domain that translocating to the nucleus to trigger the expression of downstream target genes. The IgG Fc receptor FcγRIII (CD16), recognizing blinding the Fc part of the IgG antibody of tumor cells and dissolving the cells by Ab-dependent cell-mediated cytotoxicity (ADCC), on NK cells can be cleaved by the metalloprotease ADAM17, leading to NK cell dysfunction and reduced ADCC capacity. MMPs and ADAMs can cleave ligands of the activated receptor NKG2D, such as MIC, on the surface of tumor cells, they bind to NKG2D inducing endocytosis this receptor and causing tumors to evade immune surveillance. Tumour-associated macrophages (TAM) can secret MMPs to promote tumor angiogenesis, invasion and regulate immune response. The chemokine CCL2 secreted by MMP11-overexpressing macrophages activates MAPK pathway, including Phosphorylation of ERK1/2 and JNK, through combined with its receptor CCR2, thereby promoting the migration of cancer cells by up-regulating MMP9. MMP11-expressing macrophages can also upregulate PD-L1 expression and induce immunosuppression of cancer cells. MMP2 and MMP9 released by mast cells (MCs) can promote tumor angiogenesis and tumor invasiveness, respectively. MMP2 as a physiological TLR2 ligand can specifically trigger TLR2 and then increase OX40 ligand (OX40L), which interacted with OX40, on dendritic cells (DCs) to drive T cell responses leading to modulation of immune responses.

Immune cells in the TME play an important role in tumorigenesis and possess tumor-antagonistic or tumor-promoting functions. Although anti-tumor immune cells in the TME tend to target and kill cancer cells in the early stages of tumorigenesis, cancer cells appear to inhibit the cytotoxic function of anti-tumor immune cells in a variety of ways, resulting in immune escape (130). Tumor-associated immune cells can be divided into two types according to their function: innate immune cells and adaptive immune cells. Innate immune cells, comprised of natural killer cells, eosinophils, basophils, and phagocytes, participate in tumor suppression by directly killing tumor cells or triggering adaptive immune responses. The adaptive immune system is comprised of lymphocytes (B cells and T cells), with B cells playing a major role in the humoral immune response and T cells participating in the cellular immune response (57). MPs play an important role in promoting immune cell activity and regulating immune cell migration (58, 59). The relationship between MPs and immune cells is depicted in **Figure 4**.

##### 3.2.2.1 T cells

T cells are involved in the immune response through direct secretion of soluble cytokines or cell contact-dependent mechanisms. They also play an increasingly important role in tumor immunotherapy. T cells are complex, heterogeneous and are constantly regenerating. They can also be divided into several subpopulations according to their function. Helper T (Th) cells are central regulators of the adaptive immune response, (also known as CD4+ cells because they express CD4 on their surface). They are activated by engagement of the T-cell receptor (TCR) with the major histocompatibility complex (MHC II), which is expressed on the surface of antigen-presenting cells (APCs) (60, 61). ADAM10 and ADAM17 are expressed on the surface of resting CD4+ Th cells and are important for regulating the development and function of CD4+ Th cells (16). ADAM10/17 play crucial roles in shedding of the T-cell costimulatory receptor as well as co-inhibitory receptors (62). For instance, CD154 (CD40L) is a type II membrane co-stimulatory receptor that is expressed by all antigen-activated CD4+ Th cells with the exception of “regulatory” T cells (Tregs). Following the interaction between a T cell and an APC, CD154 expression is rapidly upregulated within a few hours and is subsequently released from the T cell surface following cleavage by ADAM10 and ADAM17 (131, 132). In addition, ADAM10 and/or ADAM17 also act on the costimulatory receptor CD137, which is expressed on CD4+ and CD8+ T cells following TCR activation (63) Lymphocyte Activation Gene-3 (LAG-3; CD223) and T-cell immunoglobulin and mucin domain-containing protein-3 (TIM-3) are both T-cell coinhibitory receptors acting as substrates for ADAM10/17 protease. The soluble form of each protein (sLAG-3 and sTIM-3, respectively) in humans are formed after proteolytic cleavage of ADAM10 and ADAM17 (15, 16). Hydrolytic cleavage of LAG-3 proximal linker peptides by ADAM10 and ADAM17 yields sLAG-3, which has been shown to bind to MHCII thereby inhibiting binding of LAG3,reducing the activation of T cells, and inducing dendritic cell maturation (133, 134). Anti-cleavage of LAG-3 can inhibit antitumor T-cell responses in mice, and sTIM-3 can impair the anti-tumor immune response in T cells by reducing the expression of Th1 cytokines in CD4 effector T cells (135). Similarly, CD8+ T cells are a subset of cells that express CD8 on the cell surface. Successful binding between naive CD8+ T cells and APCs stimulates immature T cells to become activated CD8+ T cells with cytotoxic capabilities (136). It is well known that the transmembrane glycoprotein PD-L1 is expressed on the surface of tumor cells and binds to the PD-1 receptor on the surface of immune cells to inhibit T cell proliferation, block cytokine production and inhibit T-cell survival. ADAM10 and ADAM17 were recently shown to produce different cleavage products of PD-L1 that are shed from the surface of tumor cells, leading to apoptosis of CD8+ T cells and inhibition of anti-tumor immunity (137).

##### 3.2.2.2 B cells

B cells are key cellular components of humoral immunity and play a role in immune regulation and tolerance induction through various mechanisms. Unlike T cells, B cells can respond directly to antigens. Activated B cells express both MHC class I and class II molecules on the cell surface; therefore, they can present intracellular and extracellular antigens to CD4+ Th and CD8+ T cytotoxic lymphocytes (64, 65). In particular, marginal zone B (MZB) cells located in the spleen express high levels of CD80/86 costimulatory molecules leading to activation of T cells (66). Notch2 signaling is required for the development of MZB cells, which play an important role in antigen trafficking and presentation. During the development of MZB, Notch2 heterodimers bind to ligands such as DLL1 on stromal cells and APCs, which initiates an unknown metalloproteinase hydrolytic receptor resulting in the release of the Notch intracellular domain that translocate to the nucleus and triggers the expression of downstream target genes (16, 138, 139). The unknown metalloproteinase may be ADAM10. Taok3 transports ADAM10 to the surface of immature B cells, which then promote the development of immature B cells into MZB cells (16, 76, 140). In addition, studies have shown that glioma cells produce ADAM10 upon activation, and ADAM10 can induce the development of regulatory B cells (Bregs) by converting latency associated peptide (LAP) into TGF-β in B cells. Bregs not only exert immunosuppressive effects by inhibiting the activity of CD8+ T cells, but also have the ability to induce the production of Tregs, which play an important role in the evasion of tumor cells from immune surveillance (77).

##### 3.2.2.3 NK cells

Natural killer (NK) cells are specialized immune effector cells that are able to kill tumor cells and are the main source of cytokines and chemokines such as interferon (IFN)-γ and TNF-α, which regulate the function of lymphocytes and enhance the antigen-specific T-cell response (78). NK cells express an IgG Fc receptor FcγRIII (CD16). Activated NK cells can effectively recognize the Fc part of the IgG antibody that binds to tumor cells and dissolve cells by antibody-dependent cell-mediated cytotoxicity (ADCC) (141, 142). Notably, the CD16 molecule can be cleaved from the surface of activated NK cells by ADAM17, and inhibition of ADAM17 impairs the exocytotic abscission of CD16 and CD62L, which significantly increases intracellular levels of TNF-α and IFN-γ (143–145). In addition, the critical interaction between activating receptors on NK cells and MHC-I molecules (MIC) is important to prevent autoimmune destruction and facilitate evasion of immune surveillance by NK cells (146). For example, MMPs and ADAMS can cleave ligands of the activated receptor NKG2D from the surface of tumor cells. The soluble forms of these lysed proteins bind to NKG2D and induce endocytosis and degradation of this receptor, causing tumors to evade surveillance (147). Overall, there are multiple substrates lysed by ADAM17 that are associated with diverse effects on NK cells.

##### 3.2.2.4 Macrophages

Among the immune inflammatory cells in the TME, macrophages are one of the most common (148). Although macrophages have anti-tumor effects as immune cells, experimental and clinical evidence suggests that tumor-associated macrophages (TAM) contribute to cancer initiation and malignant progression, and high levels of TAMs are associated with poor prognosis and reduced overall survival (44, 149–151). Activated macrophages include M1 and M2 subtypes. M1 macrophages can kill tumor cells, while M2 macrophages mainly play a role in promoting tumor growth. However, most of the macrophages in tumor tissues have the phenotype and function of M2 macrophages. In a variety of cancers, TAMs have been found to promote tumor angiogenesis and invasion as well as regulation of the immune response by secreting MMPs (45). MMP regulation is closely related to the chemokines secreted by TAMs. MMP11 expression on macrophages is an independent negative prognostic factor in breast cancer. The chemokine CCL2 secreted by MMP11-overexpressing macrophages activates the MAPK pathway, inducing phosphorylation of ERK1/2 and JNK and promotion of HER2+ breast cancer cell migration facilitated by the upregulation of MMP9. Additionally, MMP11-expressing macrophages play a role in promoting tumor through *via* the upregulation of PD-L1 expression and inducing immunosuppression in breast cancer cells (46). In GMB, CCL5 (derived from glioma-associated microglia/brain macrophages [GAMs]) enhances glioma cell invasiveness through a novel calcium-dependent MMP2 signaling pathway (47). SLIT2 has been found to be functionally deficient in breast cancer. Mechanistic studies have shown that SLIT2-activated macrophages have high phagocytic capacity, are polarized into an anti-tumor M1 phenotype, and inhibit tumor fibrosis by activating MMP13 secreted by macrophages (48). Although evidence has suggested that MT-MMPs are expressed in primary brain tumors as effective mediators of tumor cell infiltration into central nervous system tissues, recent studies have revealed that glioma cells, rather than macrophages/microglia, are the main source of MT-MMPs (152).

##### 3.2.2.5 Mast cells

In the TME, MCs have both pro-tumor and anti-tumor properties. Once activated and degranulated, they recruit immune system cells to coordinate the anti-tumor immune response. However, their presence may contribute to tumor progression by releasing vascular endothelial growth factor to support MMP9 degradation of the ECM (153, 154). In prostate cancer, infiltrating MCs can reduce androgen receptor (AR) transcription and increase the aggressiveness of prostate cancer cells by increasing MMP9 expression (155). The invasive ability of bladder cancer cells is enhanced by upregulation of estrogen receptor β(ERβ) expression in both MCs and bladder cancer cells, resulting in increased signaling related to CCL2, CCR2, EMT, and MMP9 (156). MC progenitors may spontaneously produce MMP9 during local tissue migration, and stem cell factors can down-regulate mast cell motility by reducing MMP9 production (129). In addition, MMP2 and MMP9 released by MCs can promote angiogenesis and tumor invasiveness, respectively (157).

##### 3.2.2.6 Dendritic cells

Dendritic cells (DC)are APCs that can extract, process and present endogenous antigens to T and B lymphocytes (158, 159) Although DCs do not have the ability to kill tumor cells directly, they play a crucial role in the immune system. Recent studies have shown that in solid tumors, of the number of infiltrating DCs is directly proportional to prognosis, and DC-based vaccines have been applied in the study of tumor immunotherapy (49, 160, 161). Most DCs are in an immature state with strong antigen-phagocytosis ability. They eventually and evolve into mature DCs when they ingest antigens or are stimulated. During maturation, DCs migrate from antigen-exposed peripheral tissues into secondary lymphoid organs, where they present antigen peptides on the surface of MHC molecules to antigen-specific cognate responder T cells through the TCR, which stimulates immune responses (162, 163). The ability of mature DCs to migrate to secondary lymphoid tissues requires expression of a collagenase type IV protein, such as MMP9 (164). In a study of cervical cancer, monocyte-derived cells maintained MMP9 expression during differentiation into immature and phenotypically mature DCs (165). OX40 ligand (OX40L), which is expressed on DCs and modulated by molecules such as toll-like receptor 2 (TLR2), is a key costimulatory molecule that primes Th cells (166). MMP2 is a physiological TLR2 ligand/agonist that specifically triggers TLR2, leading to increased cytokine production and OX40L on DCs through activating components of the canonical NF-kB pathway, which results in modulation of immune responses (167). Additionally, ADAM23 expression on DCs partially governs antigen-presentation capacities to responder CD4+ T cells. Knockdown of ADAM23 in murine BMDCs did not alter the maturation profile of DCs but markedly depressed the activation, proliferation and total levels of cytokine production in CD4+ T cells, such as IL-2, IFN-γ, IL-4, and IL-17 (168). Notably, DCs have podosomes that can degrade the ECM and are proposed to be involved in cell migration (169). The podosome-related domains contain MMP14, which generates guidance tunnels within collagen gels in endothelial cells, and cancer cells use a similar mechanism as they move through the matrix (51).

#### 3.2.3 Immunomodulatory substances associated with MPs

Cytokines are proteins secreted by immune and related cells that mediate and regulate immune processes (170). According to their structure and function, cytokines can be generally divided into interleukins, interferons, TNFs, colony-stimulating factors, chemokines, and growth factors. Cytokines coordinate the interaction between the TME and tumor immune cells, and their release can inhibit or promote tumor development (57). The interaction between cytokines and MPs plays an important role in regulating the TME. Inflammatory cytokines generally upregulate the expression, secretion and activation of MPs in immune cells (11, 171, 172). In addition, they are shed as substrates or become active after cleavage by MPs leading to various immune-inflammatory responses in multiple cancers (12, 173, 174).

A variety of cytokines derived from tumor cells, including TGF-β, EGF, HGF and TNF-α, mediate the expression of many MPs. The most important of these is MMP9, which is elevated in serum and tissues associated with tumors, and is involved in the degradation of the ECM to facilitate the migration of immune cells in cancer (11). A study on breast cancer showed that MMP9 was secreted predominantly by fibroblasts, and its expression in tumor fibroblasts is regulated by multiple cytokines and complex cellular signaling pathways (175). Interleukin, as one of the most widely studied cytokines, is associated with the occurrence and development of cancer. Various cell sources, receptors and signaling pathways determine that interleukins have pleiotropic effects in cancers, including participating in immune responses through interaction with MPs (173, 176). The members can be divided into several protein families according to their structural homology. Biochemical and cell-based assays suggest that IL-2 is subject to proteolytic processing by neutrophil MMP9. The proliferation of IL-2-dependent cells, including primary human regulatory T cells, significantly decreased after IL-2 was cleaved (173).

In addition, these cytokines must be cleaved by MPs to participate in tumor immune process. TmTNF-α, cleaved by ADAM17, generates active sTNF-α (106). IL-12 also plays a critical role in T-cell development and expansion and stimulates activated T cells and NK cells to release toxic enzymes or secrete effector cytokines in the TME, which are essential for tumor clearance (50). It was hypothesized that pro-IL-12 is mostly inactive before cleavage and switches to an active state in the TME after cleavage by MMP14. The amino acid sequence recognized by MMP14 (SGRSENIRTA) was chosen as the cleavable substrate linker. The hydrolysis efficiency of this peptide sequence is 79% for MMP14, 4% for MMP2 and 9% for MMP9. Therefore, pro-IL-12 was almost completely cleaved and activity was significantly recovered after incubation with MMP14 (174).

#### 3.2.4 Relationship between metalloproteinases and angiogenesis

Tumor blood vessels can provide oxygen and nutrients and remove waste products as well as serve as a conduit for tumor cell metastasis and immune cell infiltration. In terms of structure and function, these vessels are abnormal compared with those in non-malignant tissues, which promotes progression of cancers through impaired perfusion leading to hypoxia and low pH in the TME (30). The hypoxic microenvironment caused by impaired tumor blood perfusion can promote the invasion of tumor cells and hinder the anti-cancer effect of immune cells, which produce chemokines, cytokines, proteases and microvesicles. VEGF and inflammatory chemokines are not only major proangiogenic factors, but also immune modulators, increasing angiogenesis and immune suppression (177).

To date, several types of tumor angiogenesis have been reported, including sprouting angiogenesis and vasculogenic mimicry (VM) (178). Sprouting angiogenesis is achieved by the upregulation of various hydrolases such as MPs and tissue plasmin activators in the vascular basement membrane, which leads to degradation and remodeling of the basement membrane and the ECM (**Figure 4**) (179). MMPs secreted by CAFs degrade the basement membrane of vascular endothelial cells and release VEGF bound to the extracellular matrix to initiate angiogenesis. For example, in pancreatic neuroendocrine tumors, increased secretion of MMP9 releases sequestered VEGF from the matrix, which switches vascular quiescence to active angiogenesis (31). In lung cancer cells, inhibition of MMP2 activity reduces its interaction with integrin-AVB3, and inhibits the expression of VEGF mediated by downstream PI3K/AKT signaling, leading to decreased angiogenesis (32).

VM is a newer model for invasive tumors to form new blood vessels, which provides blood supply for tumor growth (33). Studies have shown that the initial hypoxic environment of solid tumors is inseparable from VM, and that hypoxia is closely related to the expression and activity of MMPs (34, 35, 180). Hypoxia-inducible factor-1α (HIF-1α) has been shown to directly regulate the expression of MMP14, MMP9 and MMP2 (36, 180, 181). In the early stage of VM formation, MMPs dissolve ECM adhesion proteins and connexins, leading to the release of individual epithelial cells from the epithelium. Some cell fragments then initiate signal transduction pathways, leading to extensive changes in gene transcription. MMP2, activated by MMP14, causes the lysis of Ln5γ2 (laminin) and promotes the formation of VM ducts. In addition, cell protrusions termed invasive pseudopodia, aggregate the proteolytic enzymes MMP2, MMP9, and MMP14 at their leading edge where they degrade collagen and the ECM basement membrane (182). However, other studies have shown that MMP2 and MMP9 are upregulated in cancer cells through a HIF1 -dependent mechanism, whereas MMP14 is upregulated in a HIF2-dependent manner, and their enhanced activity is due to increased expression of HIF-dependent urokinase-type plasminogen activator surface receptors (183).

## 4 Recent advances and future trends in application targeting MPs in tumors diagnosis and immunotherapy

### 4.1 The crucial role of metalloproteinases in cancers diagnosis

As MPs have been found to play an important role in the occurrence and development of tumors, several diagnostic methods involving MPs have emerged in recent years. Various molecular imaging techniques have been used in cancer diagnosis to show the activity of MPs *in vivo*. For the past decade or so, MP imaging has been limited to optical imaging (OIM), positron emission tomography (PET), single photon emission computed tomography (SPECT), and magnetic resonance imaging (MRI), all of which have been inadequate in quantifying MP expression levels (184). Recently, a method has been developed for that precise quantification of MT1-MMP in cancer tissue sections using metal clusters composed of intrinsic red fluorescence and a specific mass signal. MMP14 can be directly observed *via* optical fluorescence microscopy and quantified by mass spectrometry 2D imaging (MSI) (185).

Highly selective fluorescent nanoprobes have also been developed to improve the diagnostic accuracy in early and metastatic cancers. MMP-2-responsive nanoprobes were prepared by immobilizing fluorescent fusion proteins, which consists of a fluorescent mCherry protein with a cell penetrating peptide (CPP) moiety with MMP-2 cleavage site, on nickel ferrite nanoparticles *via* the His-tag nickel chelation mechanism. The high selectivity of nanoprobes is due to the steric hindrance effect between nanoprobes and MMPs formed by hiding the cleavage site of MMP-2 substrates inside the system, which allows detection of soluble MMP-2 in the TME (186). Fluorescence nanoprobe technology can not only be used to accurately diagnose distant lymph node metastasis, but also as a prognostic tool for cancer treatment after treatment with photodynamic therapy (PDT) treatment (187). Rapid diagnosis during surgery has become an indispensable tool in cancer diagnostics. MP-mediated fluorescence nanoprobe technology has been proposed as a rapid and accurate method to assist decision-making during surgery (188).

### 4.2 Metalloproteinases inhibitors can be a potential partner for combination therapy in cancer immunotherapy

In view of the role of MPs in cancer immune regulation, it is conceivable that MPs can play a pivotal role in immunotherapy. The main immunotherapy modalities currently available are immune checkpoint blockade (ICB) therapy, chimeric antigen receptor (CAR)-T cell therapy, and cancer vaccines. Notably, the role of MP inhibitors or activators in different immunotherapy modalities is diverse. A variety of broad-spectrum MP inhibitors have emerged in clinical trials. However, due to the non-specificity of drugs and the complex role of MPs in immune regulation, MP inhibitors have so far failed to improve survival and prognosis of patients with cancer (10, 189). Recently, it has been reported that MP inhibitors can be used in combination therapy to improve the efficacy of immunotherapy (67–69).

#### 4.2.1 The mechanism of MPs as an immunomodulator

ICB has been revolutionary for cancer treatment by suppressing immunosuppressive components in the TME including programmed cell death protein 1 and its ligand (PD-1/PD-L1) and cytotoxic T lymphocyte-associated antigen 4(CTLA-4) (190). Importantly, clinical response rates of ICB have been relatively low in some cancers, despite improved treatment outcomes; therefore, combination therapy has the potential to improve ICB therapy (191). SB-3CT, as an MMP2/9 inhibitor, has been suggested to improve the efficacy of anti-PD-1 and anti-CTLA4 treatment in mouse models of melanoma and lung cancer, as well as metastatic melanoma in the lung. SB-3CT treatment not only causes a reduction of PD-L1 expression through reducing multiple oncogenic pathways, but substantially improved immune cell infiltration and cytotoxicity of T cells in combination with anti-PD-1 treatment. In addition, the combination of SB-3CT with anti-CTLA-4 enhanced the downregulation of PD-L1 expression and increased the concentration of activated tumor-infiltrating CD8+ T cells in the tumor (67). Conversely, abundant expression of MMP2 in TME could trigger a gradual enzymatic-degradation of DOX-aTIGIT-GAB hydrogel that is composed of drugs including doxorubicin (DOX) and anti-TIGIT monoclonal antibody (aTIGIT) co-packaged in an injectable enzyme-responsive hydrogel. After being stimulated by released DOX, the immunogenic tumor recruits the infiltration of NK cells and effector T cells that could be further stimulated by the subsequently released aTIGIT to boost multilayered innate and adaptive immune responses (192).

As an emerging antitumor immunotherapy, tumor vaccines, including nucleic acid, DC-based, tumor cell, and synthetic long peptide (SLP) vaccines, have achieved notable therapeutic effects in several trials. The combination of tumor vaccines with immune checkpoint inhibition or other therapies may achieve superior therapeutic effects compared with single-agent treatment (37). Many studies in recent years have focused on the use of DC vaccines to initiate and shape an anti-tumor-specific immune response and/or boost existing spontaneous anti-tumor T-cell responses (193). However, the critical pathways by which DC-based vaccines activate effective immunity remain unknown (194). DCs from patients with melanoma have reduced expression of the cell surface inducible T-cell costimulator ligand (ICOSL), which plays an importance role in activating protective T-cell responses (195). Therefore, there is potential to improve therapeutic T-cell responses and treatment outcomes in patients with cancer through improving ICOSL expression on DCs. DCs express ADAM10 and significantly increase levels of ADAM17 after maturation, which can modulate availability of ICOSL co-stimulation during humoral immune activation by cleaving surface ICOSL (196, 197). In addition, inhibition of ADAM10/17 cleavage enzyme activity in DCs can increase surface expression of ICOSL, which yielded a vaccine with more effective anti-tumor capability (198).

CARs are synthetic receptors that enable T cells to recognize tumor-associated antigens (TAAs) independent of MHC (199). Although CAR-T cell therapy has been associated with clinical responses in subsets of B-cell leukemia or lymphoma, there are several challenges for CAR-T therapy in solid tumors and malignant hematological tumors, including tumor heterogeneity (200, 201). The development of CAR-T therapy for glioblastoma (GBM) has been limited by the scarcity and heterogeneity of GBM biomarkers. Chlorotoxin (CLTX), an acid peptide, has been studied in GBM and other neuroectodermal tumors as a method to weaken tumor cell migration and invasiveness, while exhibiting minimal cross-reaction with normal cells in the brain and elsewhere (202). CAR-T cells utilizing CLTX as the targeting domain (CLTX-CAR T cells) address two major hurdles in the way of effective immunotherapy for GBM: reduction of antigen escape and maintenance of tumor cell restriction (202). MMP2, a secreted MMP, specifically and selectively interacts with CLTX and high MMP2 expression facilitates the binding of CLTX (202–204). Accordingly, MMP2 knockdown in GBM cells substantially reduced CLTX-CAR T-cell activation and cytotoxicity (202). Additionally, MMP8 have been indicated that it was positive associated with good prognosis and survival of various cancers patients. The homing of CAR-T cells can be enhanced when CAR-T cells carrying overexpressing MMP8 because MMP8 can damage the collagen fibers surrounding the tumor (205).

#### 4.2.2 Traditional and vanguard immunomodulatory drugs

##### 4.2.2.1 Monoclonal antibodies

With a high target selectivity and favorable pharmacokinetic profiles, mAbs have shown promise for immunotherapy in cancer. These mAbs modulate the activity of these by barring access to the active site, disrupting of exosite binding and preventing protease activation (206). Selective inhibition of single MMP isoforms has been previously demonstrated, e.g., the humanized monoclonal antibody Andecaliximab (GS-5745) that selectively inhibits MMP9 and Fab 3369 acting on MMP14 (207). Structural investigation revealed that GS-5745 inhibits MMP9 by binding to pro-MMP9 and preventing MMP9 activation, whereas binding to active MMP9 allosterically inhibits its activity (208). Fab 3369, derived from a synthetic humanized Fab library, intercepts endogenous MMP14 expressed on the cell surface and inhibits ECM degradation in triple-negative breast cancer (TNBC) (35). There are a variety of mAbs that effectively inhibit ADAM17, including first-generation Administration of D1(A12), second generation mAb A9 and MED13622 (206). mAbs targeting ADAMTS family members have also been studied in inflammatory and cardiovascular diseases, but not in cancer. There are also several small molecule inhibitors in clinical development that have shown positive effects in clinical trials (207).

##### 4.2.2.2 Others

Engineered nanoparticles have also shown promise for the treatment of cancer (209, 210). O-NP, an intelligent nanocarrier, contains a cationic core and a molecule consisting of hydrophobic oleic acid, as well as a MMP9-cleavable peptide and a glutamate-rich segment (OMPE). Once exposed to MMP9 in the TME, OMPE is proteolytically processed, which leads to elimination of glutamic acid residues causing a charge reversal from anionic to cationic, which enhances endocytosis of the nanocarrier in cancer cells. When administrated systemically, this phenomenon results in efficient delivery to MMP9-overexpressing tumors (211). In addition, there is a growing body of research aimed at integrating multiple therapeutic tools into one for precise molecular sensing and site-specific cancer treatment. For instance, gold nanostars (GNS), which can be attached to MMP2 polypeptides (Ac-GPLGIAGQ) and IR-780 iodide, have been utilized for enhanced photothermal therapy (PTT)/PDT in lung cancer (212).

### 4.3 Current challenge of targeting MPs in the clinical applications

Despite a number of preclinical trials have suggested that targeting MPs can bring benefits to the diagnosis and treatment of cancer, they failed at different phases in researches, mostly because to the non-specificity of the drug and the complicated background for specific effects of MPs. While some studies have begun to test highly selective MP-targeting drugs, such as the monoclonal inhibitors against MPs mentioned above, the field is still at exploring and the efficacy and safety of this approach is not yet known. In addition, whether the addition of targeting MPs will bring some potential toxicity or immune-related adverse reactions while enhancing the efficacy of tumor immunotherapy still need to be explored with more studies in future. For example, inhibition of protease activity has been reported to produce significant joint pain and swelling, as well as myelosuppression and venous thromboembolism [7]. Remarkably, with the continuous breakthroughs in biotechnology, nanoparticles are particularly attractive as a new medium for targeting MPs for cancer diagnosis and treatment. While improving the specificity, the excellent targeting efficiency of nanoparticles is confronted with the selection of the nanocarriers, its stability and sustainability.

## 5 Conclusion and perspective

In this review, we highlighted the immunomodulatory roles of MPs in the TME including ECM remodeling, signal pathway transduction, cytokine shedding and release, and promotion of angiogenesis. MPs and some relating cleavage substrates may be prospectively used as predictive biomarker candidates of prognosis for certain cancer types; however, large, confirmatory studies are required. Emerging technologies and compounds related to MPs have been increasingly explored in cancer diagnosis and treatment. As such, it is difficult to develop highly selective drugs and nanoprobes targeted towards specific MPs. Better understanding of MP expression patterns and functions in the immunoregulation of cancer will contribute to the development of more effective therapeutic approaches for cancer diagnosis and immunotherapy. Evidence shows that combinations of biomedical technologies may be more efficient for cancer therapy compared with single agents. The new technologies based on MPs are of area constant exploration and great potential. If these technologies can be put into practice, they may provide effective strategies for the diagnosis and treatment of cancer in the future.

## Author contributions

QW and KW jointly contributed to the first draft of the article. XT provided assistance in preparing figures and table. ZL and HW revised the manuscript. All authors contributed to the article and approved the submitted version.

## References

[1] Klein T Eckhard U Dufour A Solis N Overall CM. Proteolytic cleavage–mechanisms, function, and “Omic” approaches for a near-ubiquitous posttranslational modification. Chem Rev (2018) 118:1137–68. doi: 10.1021/acs.chemrev.7b00120 29265812

[2] Saw S Weiss A Khokha R Waterhouse PD. Metalloproteases: On the watch in the hematopoietic niche. Trends Immunol (2019) 40:1053–70. doi: 10.1016/j.it.2019.09.006 31645297

[3] Kessenbrock K Plaks V Werb Z. Matrix metalloproteinases: Regulators of the tumor microenvironment. Cell (2010) 141:52–67. doi: 10.1016/j.cell.2010.03.015 20371345 PMC2862057

[4] Yamamoto K Murphy G Troeberg L. Extracellular regulation of metalloproteinases. Matrix Biol (2015) 44–46:255–63. doi: 10.1016/j.matbio.2015.02.007 25701651

[5] Prior SH Fulcher YG Koppisetti RK Jurkevich A Van Doren SR. Charge-triggered membrane insertion of matrix metalloproteinase-7, supporter of innate immunity and tumors. Structure (2015) 23:2099–110. doi: 10.1016/j.str.2015.08.013 PMC4635031 26439767

[6] Yamamoto K Murphy G Troeberg L. Regulation of metalloproteinases in the extracellular environment: Emerging concepts. Matrix Biology (2015) 44–46:255–263. doi: 10.1016/j.matbio.2015.02.007 25701651

[7] Demaegdt H Laeremans H Backer J-PD Mosselmans S Le MT Kersemans V. Synergistic modulation of cystinyl aminopeptidase by divalent cation chelators. Biochem Pharmacol (2004) 68:893–900. doi: 10.1016/j.bcp.2004.05.046 15294452

[8] Rivera S. Metalloproteinases in nervous system function and pathology: introduction. Cell Mol Life Sci (2019) 76:3051–53. doi: 10.1007/s00018-019-03172-8 PMC11105757 31175371

[9] Hasan R MdNH R Ahmed R. In silico characterization and structural modeling of bacterial metalloprotease of family M4. J Genet Eng Biotechnol (2021) 19:25. doi: 10.1186/s43141-020-00105-y 33528696 PMC7851659

[10] Winer A Adams S Mignatti P. Matrix metalloproteinase inhibitors in cancer therapy: Turning past failures into future successes. Mol Cancer Ther (2018) 17:1147–55. doi: 10.1158/1535-7163.MCT-17-0646 PMC5984693 29735645

[11] Khokha R Murthy A Weiss A. Metalloproteinases and their natural inhibitors in inflammation and immunity. Nat Rev Immunol (2013) 13:649–65. doi: 10.1038/nri3499 23969736

[12] Sheu B-C Hsu S-M Ho H-N Lien H-C Huang S-C Lin R-H. A novel role of metalloproteinase in cancer-mediated immunosuppression. Cancer Res (2001) 61:237–42. 11196168

[13] Benoot T Piccioni E Ridder KD Goyvaerts C. TNFα and immune checkpoint inhibition: Friend or foe for lung cancer? Int J Mol Sci (2021) 17:8691. doi: 10.3390/ijms22168691 PMC8395431 34445397

[14] Najafi M Farhood B Mortezaee K. Extracellular matrix (ECM) stiffness and degradation as cancer drivers. J Cell Biochem (2019) 120:2782–90. doi: 10.1002/jcb.27681 30321449

[15] Fridman WH Pagès F Sautès-Fridman C Galon J. The immune contexture in human tumours: impact on clinical outcome. Nat Rev Cancer (2012) 12:298–306. doi: 10.1038/nrc3245 22419253

[16] Lambrecht BN Vanderkerken M Hammad H. The emerging role of ADAM metalloproteinases in immunity. Nat Rev Immunol (2018) 18:745–58. doi: 10.1038/s41577-018-0068-5 30242265

[17] Theocharis AD Skandalis SS Gialeli C Karamanos NK. Extracellular matrix structure. Advanced Drug Delivery Rev (2016) 97:4–27. doi: 10.1016/j.addr.2015.11.001 26562801

[18] Pastwińska J Walczak-Drzewiecka A Kozłowska E Harunari E Ratajewski M Dastych J. Hypoxia modulates human mast cell adhesion to hyaluronic acid. Immunologic Res (2022) 70:152–60. doi: 10.1007/s12026-021-09228-x PMC8917009 34791576

[19] Negrini D Passi A Moriondo A. The role of proteoglycans in pulmonaryedema development. Intensive Care Med (2008) 34:610–8. doi: 10.1007/s00134-007-0962-y 18264693

[20] Filipe EC Chitty JL Cox TR. Charting the unexplored extracellular matrix in cancer. Int J Exp Path (2018) 99:58–76. doi: 10.1111/iep.12269 29671911 PMC6031881

[21] Rowley AT Nagalla RR Wang S Liu WF. Extracellular matrix-based strategies for immunomodulatory biomaterials engineering. Adv Healthcare Mater (2019) 8:1801578. doi: 10.1002/adhm.201801578 PMC7568845 30714328

[22] Lambert J Edwards DR. Analysis of ADAMTS effects on cell adhesion and migration. In: Apte SS, editor. ADAMTS proteases. methods in molecular biology. New York, NY: Springer New York (2020). p. 179–93. doi: 10.1007/978-1-4939-9698-8_15 31463912

[23] Duffy M McGowan P Gallagher W. Cancer invasion and metastasis: changing views. J Pathol (2008) 214:283–93. doi: 10.1002/path.2282 18095256

[24] Cerdà-Costa N Xavier Gomis-Rüth F. Architecture and function of metallopeptidase catalytic domains: Metallopeptidase catalytic domains. Protein Sci (2014) 23:123–44. doi: 10.1002/pro.2400 PMC3926739 24596965

[25] Sun D Zhang Y Qi Y Zhou X Lv G. Prognostic significance of MMP-7 expression in colorectal cancer: A meta-analysis. Cancer Epidemiol (2015) 39:135–42. doi: 10.1016/j.canep.2015.01.009 25677090

[26] Jiang H Li H. Prognostic values of tumoral MMP2 and MMP9 overexpression in breast cancer: a systematic review and meta-analysis. BMC Cancer (2021) 21:149. doi: 10.1186/s12885-021-07860-2 33568081 PMC7877076

[27] Alabi RO Lora J Celen AB Maretzky T Blobel CP. Analysis of the conditions that affect the selective processing of endogenous Notch1 by ADAM10 and ADAM17. IJMS (2021) 22:1846. doi: 10.3390/ijms22041846 33673337 PMC7918056

[28] Loganathan SK Schleicher K Malik A Quevedo R Langille E Teng K. Rare driver mutations in head and neck squamous cell carcinomas converge on NOTCH signaling. Science (2020) 367:1264–9. doi: 10.1126/science.aax0902 32165588

[29] Amar S Minond D Fields GB. Clinical implications of compounds designed to inhibit ECM-modifying metalloproteinases. Proteomics (2017) 17:1600389. doi: 10.1002/pmic.201600389 28613012

[30] Fukumura D Kloepper J Amoozgar Z Duda DG Jain RK. Enhancing cancer immunotherapy using antiangiogenics: opportunities and challenges. Nat Rev Clin Oncol (2018) 15:325–40. doi: 10.1038/nrclinonc.2018.29 PMC5921900 29508855

[31] Bergers G Brekken R McMahon G Vu TH Itoh T Tamaki K. Matrix metalloproteinase-9 triggers the angiogenic switch during carcinogenesis. Nat Cell Biol (2000) 2:737–44. doi: 10.1038/35036374 PMC2852586 11025665

[32] Chetty C Lakka SS Bhoopathi P Rao JS. MMP-2 alters VEGF expression *via* αVβ3 integrin-mediated PI3K/AKT signaling in A549 lung cancer cells. Int J Cancer (2009) 127:1081–95. doi: 10.1002/ijc.25134 PMC2891576 20027628

[33] Luo Q Wang J Zhao W Peng Z Liu X Li B. Vasculogenic mimicry in carcinogenesis and clinical applications. J Hematol Oncol (2020) 13:19. doi: 10.1186/s13045-020-00858-6 32169087 PMC7071697

[34] Ma T Wang L Wang J Liu Y Chen Y He H. Hypoxia-induced cleavage of soluble ephrinA1 from cancer cells is mediated by MMP-2 and associates with angiogenesis in oral squamous cell carcinoma. OTT 2019) 12:8491–9. doi: 10.2147/OTT.S213252 PMC6799903 31686863

[35] Ling B Watt K Banerjee S Newsted D Truesdell P Adams J. A novel immunotherapy targeting MMP-14 limits hypoxia, immune suppression and metastasis in triple-negative breast cancer models. Oncotarget (2017) 8:58372–85. doi: 10.18632/oncotarget.17702 PMC5601659 28938563

[36] Li H Huang H Cui Y Li W Zhang S Chen Y. Study on the mechanism of capillary leakage caused by hypoxia-inducible factor-1α through inducing high expression of matrix metalloproteinase-9. J Oncol (2021) 2021:1–12. doi: 10.1155/2021/9130650 PMC8463177 34567119

[37] Peng M Mo Y Wang Y. Neoantigen vaccine: an emerging tumor immunotherapy. Mol Cancer (2019) 18:128. doi: 10.1186/s12943-019-1055-6 31443694 PMC6708248

[38] de Almeida LGN Thode H Eslambolchi Y Chopra S Young D Gill S. Matrix metalloproteinases: From molecular mechanisms to physiology, pathophysiology, and pharmacology. Pharmacol Rev (2022) 74:712–68. doi: 10.1124/pharmrev.121.000349 35738680

[39] Schwegmann K Bettenworth D Hermann S Faust A Poremba C Foell D. Detection of early murine colorectal cancer by MMP-2/-9–guided fluorescence endoscopy. Inflammatory Bowel Dis (2016) 22:82–91. doi: 10.1097/MIB.0000000000000605 26457379

[40] Gong L Wu D Zou J Chen J Chen L Chen Y. Prognostic impact of serum and tissue MMP-9 in non-small cell lung cancer: a systematic review and meta-analysis. Oncotarget (2016) 7:18458–68. doi: 10.18632/oncotarget.7607 PMC4951301 26918342

[41] Carey P Low E Harper E Stack MS. Metalloproteinases in ovarian cancer. IJMS (2021) 22:3403. doi: 10.3390/ijms22073403 33810259 PMC8036623

[42] Derynck R Turley SJ Akhurst RJ. TGFβ biology in cancer progression and immunotherapy. Nat Rev Clin Oncol (2021) 18:9–34. doi: 10.1038/s41571-020-0403-1 32710082 PMC9721352

[43] Batlle E Massagué J. Transforming growth factor-β signaling in immunity and cancer. Immunity (2019) 50:924–40. doi: 10.1016/j.immuni.2019.03.024 PMC7507121 30995507

[44] Qian B-Z Pollard JW. Macrophage diversity enhances tumor progression and metastasis. Cell (2010) 141:39–51. doi: 10.1016/j.cell.2010.03.014 20371344 PMC4994190

[45] Chen Y Song Y Du W Gong L Chang H Zou Z. Tumor-associated macrophages: an accomplice in solid tumor progression. J Biomed Sci. (2019) 26:78. doi: 10.1186/s12929-019-0568-z PMC6800990 31629410

[46] Kang SU Cho SY Jeong H. Matrix metalloproteinase 11 (MMP11) in macrophages promotes the migration of HER2-positive breast cancer cells and monocyte recruitment through CCL2–CCR2 signaling. Lab Invest (2022) 102:376–90. doi: 10.1038/s41374-021-00699-y 34775491

[47] Yu-Ju Wu C Chen C-H Lin C-Y Feng L-Y Lin Y-C Wei K-C. CCL5 of glioma-associated microglia/macrophages regulates glioma migration and invasion *via* calcium-dependent matrix metalloproteinase 2. Neuro-Oncology (2020) 22:253–66. doi: 10.1093/neuonc/noz189 PMC7032635 31593589

[48] Ahirwar DK Charan M Mishra S Verma AK Shilo K Ramaswamy B. Slit2 inhibits breast cancer metastasis by activating M1-like phagocytic and antifibrotic macrophages. Cancer Res (2021) 81:5255–67. doi: 10.1158/0008-5472.CAN-20-3909 PMC8631742 34400395

[49] Gilboa E. DC-Based cancer vaccines. J Clin Invest (2007) 117:1195–203. doi: 10.1172/JCI31205 PMC1857263 17476349

[50] Heinzel FP Rerko RM Ling P Hakimi J Schoenhaut DS. Interleukin 12 is produced in vivo during endotoxemia and stimulates synthesis of gamma interferon. Infect Immun (1994) 62:4244–49. doi: 10.1128/iai.62.10.4244-4249.1994 PMC303101 7927680

[51] Gawden-Bone C Zhou Z King E Prescott A Watts C Lucocq J. Dendritic cell podosomes are protrusive and invade the extracellular matrix using metalloproteinase MMP-14. J Cell Sci (2010) 123:1427–37. doi: 10.1242/jcs.056515 PMC2858019 20356925

[52] Herrlich P Herrlich A. ADAM metalloprotease-released cancer biomarkers. Trends Cancer (2017) 3:482–90. doi: 10.1016/j.trecan.2017.05.001 28718403

[53] Serrano-Coll H Acevedo-Saenz L Cardona-Castro N. A hypothetical role for notch signaling pathway in immunopathogenesis of leprosy. Med Hypotheses (2017) 109:162–9. doi: 10.1016/j.mehy.2017.10.009 29150278

[54] Sierra RA Thevenot P Raber PL Cui Y Parsons C Ochoa AC. Rescue of notch-1 signaling in antigen-specific CD8+ T cells overcomes tumor-induced T-cell suppression and enhances immunotherapy in cancer. Cancer Immunol Res (2014) 2:800–11. doi: 10.1158/2326-6066.CIR-14-0021 PMC4125513 24830414

[55] Christian L. The ADAM family: Insights into notch proteolysis. Fly (2012) 6:30–4. doi: 10.4161/fly.18823 22513479

[56] Pacheco-Fernandez N Pakdel M Blank B Sanchez-Gonzalez I Weber K Tran ML. Nucleobindin-1 regulates ECM degradation by promoting intra-golgi trafficking of MMPs. J Cell Biol (2020) 219:e201907058. doi: 10.1083/jcb.201907058 32479594 PMC7401813

[57] Hinshaw DC Shevde LA. The tumor microenvironment innately modulates cancer progression. Cancer Res (2019) 79:4557–66. doi: 10.1158/0008-5472.CAN-18-3962 PMC6744958 31350295

[58] McMahon M Ye S Pedrina J Dlugolenski D Stambas J. Extracellular matrix enzymes and immune cell biology. Front Mol Biosci (2021) 8:703868. doi: 10.3389/fmolb.2021.703868 34527702 PMC8436118

[59] Vaday GG Lider O. Extracellular matrix moieties, cytokines, and enzymes: dynamic effects on immune cell behavior and inflammation. J Leukoc Biol (2000) 67:149–59. doi: 10.1002/jlb.67.2.149 10670574

[60] Holling TM Schooten E van Den Elsen PJ. Function and regulation of MHC class II molecules in T-lymphocytes: of mice and men. Hum Immunol (2004) 65:282–90. doi: 10.1016/j.humimm.2004.01.005 15120183

[61] Carreño LJ Bueno SM Bull P Nathenson SG Kalergis AM. The half-life of the T-cell receptor/peptide?major histocompatibility complex interaction can modulate T-cell activation in response to bacterial challenge. Immunology (2007) 121:227–37. doi: 10.1111/j.1365-2567.2007.02561.x PMC2265936 17313485

[62] Sezin T Selvakumar B Scheffold A. The role of a disintegrin and metalloproteinase (ADAM)-10 in T helper cell biology. Biochimica et Biophysica Acta (BBA) - Molecular Cell Research (2022) 1869:119192. doi: 10.1016/j.bbamcr.2021.119192 34982961

[63] Seidel J Leitzke S Ahrens B Sperrhacke M Bhakdi S Reiss K. Role of ADAM10 and ADAM17 in regulating CD137 function. IJMS (2021) 22:2730. doi: 10.3390/ijms22052730 33800462 PMC7962946

[64] Ghosh D Jiang W Mukhopadhyay D Mellins ED. New insights into b cells as antigen presenting cells. Curr Opin Immunol (2021) 70:129–37. doi: 10.1016/j.coi.2021.06.003 34242927

[65] Zhang Y Morgan R Podack ER Rosenblatt J. B cell regulation of anti-tumor immune response. Immunol Res (2013) 57:115–24. doi: 10.1007/s12026-013-8472-1 24293009

[66] Attanavanich K Kearney JF. Marginal zone, but not follicular b cells, are potent activators of naive CD4 T cells. J Immunol (2004) 172:803–11. doi: 10.4049/jimmunol.172.2.803 14707050

[67] Ye Y Kuang X Xie Z. Small-molecule MMP2/MMP9 inhibitor SB-3CT modulates tumor immune surveillance by regulating PD-L1. Genome Med (2020) 12:83. doi: 10.1186/s13073-020-00780-z 32988398 PMC7523356

[68] Zhou Q Gil-Krzewska A Peruzzi G Borrego F. Matrix metalloproteinases inhibition promotes the polyfunctionality of human natural killer cells in therapeutic antibody-based anti-tumour immunotherapy. Clin Exp Immunol (2013) 173:131–9. doi: 10.1111/cei.12095 PMC3694543 23607800

[69] Liu Y Chen X-G Yang P-P Qiao Z-Y Wang H. Tumor microenvironmental pH and enzyme dual responsive polymer-liposomes for synergistic treatment of cancer immuno-chemotherapy. Biomacromolecules (2019) 20:882–92. doi: 10.1021/acs.biomac.8b01510 30621390

[70] Chang W-J. Inflammation-related factors predicting prognosis of gastric cancer. WJG (2014) 20:4586. doi: 10.3748/wjg.v20.i16.4586 24782611 PMC4000495

[71] Reiss K Saftig P. The “A disintegrin and metalloprotease” (ADAM) family of sheddases: Physiological and cellular functions. Semin Cell Dev Biol (2009) 20:126–37. doi: 10.1016/j.semcdb.2008.11.002 19049889

[72] Balkwill F. TNF-α in promotion and progression of cancer. Cancer Metastasis Rev (2006) 25:409–16. doi: 10.1007/s10555-006-9005-3 16951987

[73] Horiuchi T Mitoma H Harashima S Tsukamoto H Shimoda T. Transmembrane TNF-a: structure, function and interaction with anti-TNF agents. Rheumatology (Oxford) (2010) 49:1215–28. doi: 10.1093/rheumatology/keq031 PMC2886310 20194223

[74] Balkwill F. Tumour necrosis factor and cancer. Nat Rev Cancer (2009) 9:361–71. doi: 10.1038/nrc2628 19343034

[75] Gordon WR Arnett KL Blacklow SC. The molecular logic of notch signaling – a structural and biochemical perspective. J Cell Sci (2008) 121:3109–19. doi: 10.1242/jcs.035683 PMC2696053 18799787

[76] Hammad H Vanderkerken M Pouliot P. Transitional b cells commit to marginal zone b cell fate by Taok3-mediated surface expression of ADAM10. Nat Immunol (2017) 18:313–20. doi: 10.1038/ni.3657 28068307

[77] Ye Z He H Wang H Li W Luo L Huang Z. Glioma-derived ADAM10 induces regulatory b cells to suppress CD8+ T cells. PloS One (2014) 9:e105350. doi: 10.1371/journal.pone.0105350 25127032 PMC4134303

[78] Liu S Galat V Galat Y Lee YKA Wainwright D Wu J. NK cell-based cancer immunotherapy: from basic biology to clinical development. J Hematol Oncol (2021) 14:7. doi: 10.1186/s13045-020-01014-w 33407739 PMC7788999

[79] ADAM TS ADAMTS Family Proteins and Snake Venom Metalloproteinases. A structural overview. Toxins (2016) 8:155. doi: 10.3390/toxins8050155 27196928 PMC4885070

[80] Pluda S Mazzocato Y Angelini A. Peptide-based inhibitors of ADAM and ADAMTS metalloproteinases. Front Mol Biosci (2021) 8:703715. doi: 10.3389/fmolb.2021.703715 34368231 PMC8335159

[81] Kelwick R Desanlis I Wheeler GN Edwards DR. The ADAMTS (A disintegrin and metalloproteinase with thrombospondin motifs) family. Genome Biol (2015) 16:113. doi: 10.1186/s13059-015-0676-3 26025392 PMC4448532

[82] Apte SS. A disintegrin-like and metalloprotease (Reprolysin-type) with thrombospondin type 1 motif (ADAMTS) superfamily: Functions and mechanisms. J Biol Chem (2009) 284:31493–7. doi: 10.1074/jbc.R109.052340 PMC2797218 19734141

[83] Visse R Nagase H. Matrix metalloproteinases and tissue inhibitors of metalloproteinases: Structure, function, and biochemistry. Circ Res (2003) 92:827–39. doi: 10.1161/01.RES.0000070112.80711.3D 12730128

[84] Nagase H Visse R Murphy G. Structure and function of matrix metalloproteinases and TIMPs. Cardiovasc Res (2006) 69:562–73. doi: 10.1016/j.cardiores.2005.12.002 16405877

[85] Cauwe B Van den Steen PE Opdenakker G. The biochemical, biological, and pathological kaleidoscope of cell surface substrates processed by matrix metalloproteinases. Crit Rev Biochem Mol Biol (2007) 42:113–85. doi: 10.1080/10409230701340019 17562450

[86] Cui N Hu M Khalil RA. Biochemical and biological attributes of matrix metalloproteinases.,” progress in molecular biology and translational science. Elsevier (2017) p. 1–73. doi: 10.1016/bs.pmbts.2017.02.005 PMC5430303 28413025

[87] Hadler-Olsen E Fadnes B Sylte I Uhlin-Hansen L Winberg J-O. Regulation of matrix metalloproteinase activity in health and disease: Regulation of MMP activity. FEBS J (2011) 278:28–45. doi: 10.1111/j.1742-4658.2010.07920.x 21087458

[88] Nyante SJ Wang T Tan X Ozdowski EF Lawton TJ. Quantitative expression of MMPs 2, 9, 14, and collagen IV in LCIS and paired normal breast tissue. Sci Rep (2019) 9:13432. doi: 10.1038/s41598-019-48602-6 31530842 PMC6748975

[89] Zucker S Vacirca J. Role of matrix metalloproteinases (MMPs) in colorectal cancer. Cancer Metastasis Rev (2004) 23:101–17. doi: 10.1023/A:1025867130437 15000152

[90] Edwards D Handsley M Pennington C. The ADAM metalloproteinases. Mol Aspects Med (2008) 29:258–89. doi: 10.1016/j.mam.2008.08.001 PMC7112278 18762209

[91] Camodeca C Cuffaro D Nuti E Rossello A. ADAM metalloproteinases as potential drug targets. CMC (2019) 26:2661–89. doi: 10.2174/0929867325666180326164104 29589526

[92] Seegar TC Blacklow SC. Domain integration of ADAM family proteins: Emerging themes from structural studies. Exp Biol Med (Maywood) (2019) 244:1510–9. doi: 10.1177/1535370219865901 PMC6920674 31333048

[93] Lu X Lu D Scully M Kakkar V. ADAM proteins- therapeutic potential in cancer. Curr Cancer Drug Targets (2008) 8:720–32. doi: 10.2174/156800908786733478 19075595

[94] Rogmans C Kuhlmann JD Hugendieck G Link T Arnold N Weimer JP. ADAM17–a potential blood-based biomarker for detection of early-stage ovarian cancer. Cancers (2021) 13:5563. doi: 10.3390/cancers13215563 34771725 PMC8583642

[95] Yoneyama T Gorry M Sobo-Vujanovic A Lin Y Vujanovic L Gaither-Davis A. ADAM10 sheddase activity is a potential lung-cancer biomarker. J Cancer (2018) 9:2559–70. doi: 10.7150/jca.24601 PMC6036891 30026855

[96] Cal S López-Otín C. ADAMTS proteases and cancer. Matrix Biol (2015) 44–46:77–85. doi: 10.1016/j.matbio.2015.01.013 25636539

[97] Binder MJ McCoombe S Williams ED McCulloch DR Ward AC. The extracellular matrix in cancer progression: Role of hyalectan proteoglycans and ADAMTS enzymes. Cancer Lett (2017) 385:55–64. doi: 10.1016/j.canlet.2016.11.001 27838414

[98] Zhang X Yang W Chen K Zheng T Guo Z Peng Y. The potential prognostic values of the ADAMTS-like protein family: an integrative pan-cancer analysis. Ann Transl Med (2021) 9:1562–2. doi: 10.21037/atm-21-4946 PMC8576672 34790768

[99] Zhang Y Hu K Qu Z Xie Z Tian F. ADAMTS8 inhibited lung cancer progression through suppressing VEGFA. Biochem Biophys Res Commun (2022) 598:1–8. doi: 10.1016/j.bbrc.2022.01.110 35149432

[100] Rabadán R Mohamedi Y Rubin U Chu T Alghalith AN Elliott O. Identification of relevant genetic alterations in cancer using topological data analysis. Nat Commun (2020) 11:3808. doi: 10.1038/s41467-020-17659-7 32732999 PMC7393176

[101] Karabela SP Kairi CA Magkouta S Psallidas I Moschos C Stathopoulos I. Neutralization of tumor necrosis factor bioactivity ameliorates urethane-induced pulmonary oncogenesis in mice. Neoplasia (2011) 13:1143–51. doi: 10.1593/neo.111224 PMC3257189 22241960

[102] Guo X-F. *TNF-α-308* polymorphism and risk of digestive system cancers: A meta-analysis. WJG (2013) 19:9461. doi: 10.3748/wjg.v19.i48.9461 24409077 PMC3882423

[103] Ma L Zhao J Li T He Y Wang J Xie L. Association between tumor necrosis factor-alpha gene polymorphisms and prostate cancer risk: a meta-analysis. Diagn Pathol (2014) 9:74. doi: 10.1186/1746-1596-9-74 24666463 PMC3977697

[104] Cruceriu D Baldasici O Balacescu O Berindan-Neagoe I. The dual role of tumor necrosis factor-alpha (TNF-α) in breast cancer: molecular insights and therapeutic approaches. Cell Oncol (Dordr) (2020) 43:1–18. doi: 10.1007/s13402-019-00489-1 PMC12990688 31900901

[105] Badenes M Adrain C. iRhom2 and TNF: Partners or enemies? Sci Signal (2019) 12:eaaz0444. doi: 10.1126/scisignal.aaz0444 31662485

[106] Bhattacharyya S Ghosh SS. Unfolding transmembrane TNFα dynamics in cancer therapeutics. Cytokine (2021) 137:155303. doi: 10.1016/j.cyto.2020.155303 33002738

[107] Scheller J Chalaris A Garbers C Rose-John S. ADAM17: a molecular switch to control inflammation and tissue regeneration. Trends Immunol (2011) 32:380–7. doi: 10.1016/j.it.2011.05.005 21752713

[108] Bolik J Krause F Stevanovic M Gandraß M Thomsen I Schacht S-S. Inhibition of ADAM17 impairs endothelial cell necroptosis and blocks metastasis. J Exp Med (2022) 219:e20201039. doi: 10.1084/jem.20201039 34919140 PMC8689681

[109] Zidi I Mestiri S Bartegi A Amor NB. TNF-a and its inhibitors in cancer. Med Oncol (2010) 27:185–98. doi: 10.1007/s12032-009-9190-3 19277912

[110] Chen AY Wolchok JD Bass AR. TNF in the era of immune checkpoint inhibitors: friend or foe? Nat Rev Rheumatol (2021) 17:213–23. doi: 10.1038/s41584-021-00584-4 PMC8366509 33686279

[111] Zunke F Rose-John S. The shedding protease ADAM17: Physiology and pathophysiology. Biochim Biophys Acta (BBA) - Mol Cell Res (2017) 1864:2059–70. doi: 10.1016/j.bbamcr.2017.07.001 28705384

[112] Robertson IB Horiguchi M Zilberberg L Dabovic B Hadjiolova K Rifkin DB. Latent TGF-β-binding proteins. Matrix Biol (2015) 47:44–53. doi: 10.1016/j.matbio.2015.05.005 25960419 PMC4844006

[113] Syed V. TGF-β signaling in cancer. J Cell Biochem (2016) 117:1279–87. doi: 10.1002/jcb.25496 26774024

[114] Yu Q Stamenkovic I. Cell surface-localized matrix metalloproteinase-9 proteolytically activates TGF-β and promotes tumor invasion and angiogenesis. Genes Dev (2000) 14:163–76. doi: 10.1101/gad.14.2.163 PMC316345 10652271

[115] Jabłońska-Trypuć A Matejczyk M Rosochacki S. Matrix metalloproteinases (MMPs), the main extracellular matrix (ECM) enzymes in collagen degradation, as a target for anticancer drugs. J Enzyme Inhibition Medicinal Chem (2016) 31:177–83. doi: 10.3109/14756366.2016.1161620 27028474

[116] Dong H Diao H Zhao Y Xu H Pei S Gao J. Overexpression of matrix metalloproteinase-9 in breast cancer cell lines remarkably increases the cell malignancy largely *via* activation of transforming growth factor beta/SMAD signalling. Cell Prolif (2019) 52:e12633. doi: 10.1111/cpr.12633 31264317 PMC6797518

[117] David CJ Massagué J. Contextual determinants of TGFβ action in development, immunity and cancer. Nat Rev Mol Cell Biol (2018) 19:419–35. doi: 10.1038/s41580-018-0007-0 PMC7457231 29643418

[118] Baghy K Reszegi A Tátrai P Kovalszky I. Decorin in the tumor microenvironment Tumor microenvironment. advances in experimental medicine and biology. In: Birbrair A, editor. Cham: Springer International Publishing (2020). p. 17–38. doi: 10.1007/978-3-030-48457-6_2 32845500

[119] Fu W Cai R Ma Z Li T Lei C Zhao J. TIGIT-fc as a potential therapeutic agent for fetomaternal tolerance. Front Immunol (2021) 12:649135. doi: 10.3389/fimmu.2021.649135 33841433 PMC8027249

[120] Reichrath J Reichrath S. Notch signaling in embryology and cancer: Notch signaling in cancer. Cham: Springer International Publishing (2021). doi: 10.1007/978-3-030-55031-8

[121] Stephenson NL Avis JM. Direct observation of proteolytic cleavage at the S2 site upon forced unfolding of the notch negative regulatory region. Proc Natl Acad Sci USA (2012) 109:E2757–E2765. doi: 10.1073/pnas.1205788109 23011796 PMC3478610

[122] van Tetering G van Diest P Verlaan I van der Wall E Kopan R Vooijs M. Metalloprotease ADAM10 is required for Notch1 site 2 cleavage. J Biol Chem (2009) 284:31018–27. doi: 10.1074/jbc.M109.006775 PMC2781502 19726682

[123] Marozzi M Parnigoni A Negri A Viola M Vigetti D Passi A. Inflammation, extracellular matrix remodeling, and proteostasis in tumor microenvironment. IJMS (2021) 22:8102. doi: 10.3390/ijms22158102 34360868 PMC8346982

[124] Hoshino D Koshikawa N Suzuki T Quaranta V Weaver AM Seiki M. Establishment and validation of computational model for MT1-MMP dependent ECM degradation and intervention strategies. PloS Comput Biol (2012) 8:e1002479. doi: 10.1371/journal.pcbi.1002479 22511862 PMC3325185

[125] Kumar S Das A Barai A Sen S. MMP secretion rate and inter-invadopodia spacing collectively govern cancer invasiveness. Biophys J (2018) 114:650–62. doi: 10.1016/j.bpj.2017.11.3777 PMC5985010 29414711

[126] Niland S Riscanevo AX Eble JA. Matrix metalloproteinases shape the tumor microenvironment in cancer progression. IJMS (2021) 23:146. doi: 10.3390/ijms23010146 35008569 PMC8745566

[127] Stuelten CH Zhang YE. Transforming growth factor-β: An agent of change in the tumor microenvironment. Front Cell Dev Biol (2021) 9:764727. doi: 10.3389/fcell.2021.764727 34712672 PMC8545984

[128] Ferrari N Calvo F. Tumor microenvironment: Unleashing metalloproteinases to induce a CAF phenotype. Curr Biol (2014) 24:R1009–11. doi: 10.1016/j.cub.2014.09.036 25442849

[129] Tanaka A Arai K Kitamura Y Matsuda H. Matrix metalloproteinase-9 production, a newly identified function of mast cell progenitors, is downregulated by c-kit receptor activation. Blood (1999) 94:2390–5. doi: 10.1182/blood.V94.7.2390.419k16_2390_2395 10498611

[130] Lei X Lei Y Li J-K Du W-X Li R-G Yang J. Immune cells within the tumor microenvironment: Biological functions and roles in cancer immunotherapy. Cancer Lett (2020) 470:126–33. doi: 10.1016/j.canlet.2019.11.009 31730903

[131] Yacoub D Benslimane N Al-Zoobi L Hassan G Nadiri A Mourad W. CD154 is released from T-cells by a disintegrin and metalloproteinase domain-containing protein 10 (ADAM10) and ADAM17 in a CD40 protein-dependent manner. J Biol Chem (2013) 288:36083–93. doi: 10.1074/jbc.M113.506220 PMC3861656 24189063

[132] Salti S Al-Zoobi L Darif Y Hassan GS Mourad W. CD154 resistant to cleavage from intracellular milieu and cell surface induces more potent CD40-mediated responses. J Immunol (2021) 206:1793–805. doi: 10.4049/jimmunol.2001340 33762325

[133] Li N Wang Y Forbes K Vignali KM Heale BS Saftig P. Metalloproteases regulate T-cell proliferation and effector function *via* LAG-3. EMBO J (2007) 26:494–504. doi: 10.1038/sj.emboj.7601520 17245433 PMC1783452

[134] Graydon CG Mohideen S Fowke KR. LAG3’s enigmatic mechanism of action. Front Immunol (2021) 11:615317. doi: 10.3389/fimmu.2020.615317 33488626 PMC7820757

[135] Andrews LP Somasundaram A Moskovitz JM Szymczak-Workman AL Liu C Cillo AR. Resistance to PD1 blockade in the absence of metalloprotease-mediated LAG3 shedding. Sci Immunol (2020) 5:eabc2728. doi: 10.1126/sciimmunol.abc2728 32680952 PMC7901539

[136] Xia D Hao S Xiang J. CD8 ^+^ cytotoxic T-APC stimulate central memory CD8 ^+^ T cell responses *via* acquired peptide-MHC class I complexes and CD80 costimulation, and IL-2 secretion. J Immunol (2006) 177:2976–84. doi: 10.4049/jimmunol.177.5.2976 16920933

[137] Orme JJ Jazieh KA Xie T Harrington S Liu X Ball M. ADAM10 and ADAM17 cleave PD-L1 to mediate PD-(L)1 inhibitor resistance. Oncoimmunology (2020) 9:1744980. doi: 10.1080/2162402X.2020.1744980 32363112 PMC7185206

[138] Lechner M Engleitner T Babushku T Schmidt-Supprian M Rad R Strobl LJ. Notch2-mediated plasticity between marginal zone and follicular B cells. Nat Commun (2021) 12:1111. doi: 10.1038/s41467-021-21359-1 33597542 PMC7889629

[139] Sheng Y Yahata T Negishi N Nakano Y Habu S Hozumi K. Expression of delta-like 1 in the splenic non-hematopoietic cells is essential for marginal zone b cell development. Immunol Lett (2008) 121:33–37. doi: 10.1016/j.imlet.2008.08.001 18786568

[140] Gibb DR El Shikh M Kang D-J Rowe WJ El Sayed R Cichy J. ADAM10 is essential for Notch2-dependent marginal zone b cell development and CD23 cleavage *in vivo* . J Exp Med (2010) 207:623–35. doi: 10.1084/jem.20091990 PMC2839139 20156974

[141] Wang W Erbe AK Hank JA Morris ZS Sondel PM. NK cell-mediated antibody-dependent cellular cytotoxicity in cancer immunotherapy. Front Immunol (2015) 6:368. doi: 10.3389/fimmu.2015.00368 26284063 PMC4515552

[142] Ge S Chu M Choi J Louie S Vo A Jordan SC. Imlifidase inhibits HLA antibody-mediated NK cell activation and antibody-dependent cell-mediated cytotoxicity (ADCC). In Vitro Transplant (2020) 104:1574–9. doi: 10.1097/TP.0000000000003023 32732834

[143] Zhu H Blum RH Bjordahl R. Pluripotent stem cell-derived NK cells with high-affinity non-cleavable CD16a mediate improved anti-tumor activity. Blood (2020) 135:399–410. doi: 10.1182/blood.2019000621 PMC7005364 31856277

[144] Romee R Foley B Lenvik T Wang Y Zhang B Ankarlo D. NK cell CD16 surface expression and function is regulated by a disintegrin and metalloprotease-17 (ADAM17). Blood (2013) 121:3599–608. doi: 10.1182/blood-2012-04-425397 PMC3643761 23487023

[145] Wu J Mishra HK Walcheck B. Role of ADAM17 as a regulatory checkpoint of CD16A in NK cells and as a potential target for cancer immunotherapy. J Leukoc Biol (2019) 105:1297–303. doi: 10.1002/JLB.2MR1218-501R PMC6792391 30786043

[146] Zhang C Zhang J Wei H Tian Z. Imbalance of NKG2D and its inhibitory counterparts: How does tumor escape from innate immunity? Int Immunopharmacol (2005) 5:1099–111. doi: 10.1016/j.intimp.2005.03.003 15914316

[147] Waldhauer I Goehlsdorf D Gieseke F Weinschenk T Wittenbrink M Ludwig A. Tumor-associated MICA is shed by ADAM proteases. Cancer Res (2008) 68:6368–76. doi: 10.1158/0008-5472.CAN-07-6768 18676862

[148] Fu L-Q Du W-L Cai M-H Yao J-Y Zhao Y-Y Mou X-Z. The roles of tumor-associated macrophages in tumor angiogenesis and metastasis. Cell Immunol (2020) 353:104119. doi: 10.1016/j.cellimm.2020.104119 32446032

[149] Yin S Huang J Li Z Zhang J Luo J Lu C. The prognostic and clinicopathological significance of tumor-associated macrophages in patients with gastric cancer: A meta-analysis. PloS One (2017) 12:e0170042. doi: 10.1371/journal.pone.0170042 28081243 PMC5230964

[150] Mei J Xiao Z Guo C Pu Q Ma L Liu C. Prognostic impact of tumor-associated macrophage infiltration in non-small cell lung cancer: A systemic review and meta-analysis. Oncotarget (2016) 7:34217–28. doi: 10.18632/oncotarget.9079 PMC5085150 27144518

[151] Zhang Q Liu L Gong C Shi H Zeng Y Wang X. Prognostic significance of tumor-associated macrophages in solid tumor: A meta-analysis of the literature. PloS One (2012) 7:e50946. doi: 10.1371/journal.pone.0050946 23284651 PMC3532403

[152] Giuliani C. The flavonoid quercetin induces AP-1 activation in FRTL-5 thyroid cells. Antioxidants (2019) 8:112. doi: 10.3390/antiox8050112 31035637 PMC6562732

[153] Komi DEA Redegeld FA. Role of mast cells in shaping the tumor microenvironment. Clinic Rev Allerg Immunol (2020) 58:313–25. doi: 10.1007/s12016-019-08753-w PMC7244463 31256327

[154] Heissig B Rafii S Akiyama H Ohki Y Sato Y Rafael T. Low-dose irradiation promotes tissue revascularization through VEGF release from mast cells and MMP-9–mediated progenitor cell mobilization. J Exp Med (2005) 202:739–50. doi: 10.1084/jem.20050959 PMC2212942 16157686

[155] Li L Dang Q Xie H Yang Z He D Liang L. Infiltrating mast cells enhance prostate cancer invasion via altering LncRNA-HOTAIR/PRC2-androgen receptor (AR)-MMP9 signals and increased stem/progenitor cell population. Oncotarget (2015) 6:14179–90. doi: 10.18632/oncotarget.3651 10.18632/oncotarget.3651 PMC4546459 25895025

[156] Rao Q Chen Y Yeh C-R Jie D Li L Yeh S. Recruited mast cells in the tumor microenvironment enhance bladder cancer metastasis via modulation of ERβ/CCL2/CCR2 EMT/MMP9 signals. Oncotarget (2016) 7(7):7842–55. doi: 10.18632/oncotarget.5467 PMC4884958 26556868

[157] Ribatti D Crivellato E. Mast cells, angiogenesis, and tumour growth. Biochimica et Biophysica Acta (BBA) - Molecular Basis of Disease (2012) 1822:2–8. doi: 10.1016/j.bbadis.2010.11.010 21130163

[158] Gardner A Ruffell B. Dendritic cells and cancer immunity. Trends Immunol (2016) 37:855–65. doi: 10.1016/j.it.2016.09.006 PMC5135568 27793569

[159] Ma Y Aymeric L Locher C Kroemer G Zitvogel L. The dendritic cell–tumor cross-talk in cancer. Curr Opin Immunol (2011) 23:146–52. doi: 10.1016/j.coi.2010.09.008 20970973

[160] Bol KF Schreibelt G Gerritsen WR de Vries IJ Figdor CG. Dendritic cell–based immunotherapy: State of the art and beyond. Clin Cancer Res (2016) 22:1897–906. doi: 10.1158/1078-0432.CCR-15-1399 27084743

[161] Hashemi V Farhadi S Ghasemi Chaleshtari M Seashore-Ludlow B Masjedi A Hojjat-Farsangi M. Nanomedicine for improvement of dendritic cell-based cancer immunotherapy. Int Immunopharmacol (2020) 83:106446. doi: 10.1016/j.intimp.2020.106446 32244048

[162] Hopkins RA Connolly JE. The specialized roles of immature and mature dendritic cells in antigen cross-presentation. Immunol Res (2012) 53:91–107. doi: 10.1007/s12026-012-8300-z 22450675

[163] de Winde CM Munday C Acton SE. Molecular mechanisms of dendritic cell migration in immunity and cancer. Med Microbiol Immunol (2020) 209:515–29. doi: 10.1007/s00430-020-00680-4 PMC7395046 32451606

[164] Ratzinger G Stoitzner P Ebner S Lutz MB Layton GT Rainer C. Matrix metalloproteinases 9 and 2 are necessary for the migration of langerhans cells and dermal dendritic cells from human and murine skin. J Immunol (2002) 168:4361–71. doi: 10.4049/jimmunol.168.9.4361 11970978

[165] Pahne-Zeppenfeld J Schröer N Walch-Rückheim B Oldak M Gorter A Hegde S. Cervical cancer cell-derived interleukin-6 impairs CCR7-dependent migration of MMP-9-expressing dendritic cells: Dissociation of CCR7 and mmp-9 in cervical cancer DC. Int J Cancer (2014) 134:2061–73. doi: 10.1002/ijc.28549 24136650

[166] Ito T Wang Y-H Duramad O Hori T Delespesse GJ Watanabe N. TSLP-activated dendritic cells induce an inflammatory T helper type 2 cell response through OX40 ligand. J Exp Med (2005) 202:1213–23. doi: 10.1084/jem.20051135 PMC2213234 16275760

[167] Godefroy E Gallois A Idoyaga J Merad M Tung N Monu N. Activation of toll-like receptor-2 by endogenous matrix metalloproteinase-2 modulates dendritic-Cell-Mediated inflammatory responses. Cell Reports (2014) 9:1856–70. doi: 10.1016/j.celrep.2014.10.067 PMC4336179 25466255

[168] Elizondo DM Andargie TE Marshall KM Zariwala AM Lipscomb MW. Dendritic cell expression of ADAM23 governs T cell proliferation and cytokine production through the α(v)β(3) integrin receptor. J Leukocyte Biol (2016) 100:855–64. doi: 10.1189/jlb.2HI1115-525R PMC6608068 27317750

[169] Wernimont SA Cortesio CL Simonson WTN Huttenlocher A. Adhesions ring: A structural comparison between podosomes and the immune synapse. Eur J Cell Biol (2008) 87:507–15. doi: 10.1016/j.ejcb.2008.01.011 PMC2570187 18343530

[170] Berraondo P Sanmamed MF Ochoa MC Etxeberria I Aznar MA Pérez-Gracia JL. Cytokines in clinical cancer immunotherapy. Br J Cancer (2019) 120:6–15. doi: 10.1038/s41416-018-0328-y 30413827 PMC6325155

[171] Zhang Q Liu S Parajuli KR Zhang W Zhang K Mo Z. Interleukin-17 promotes prostate cancer *via* MMP7-induced epithelial-to-mesenchymal transition. Oncogene (2017) 36:687–99. doi: 10.1038/onc.2016.240 PMC5213194 27375020

[172] Wu J Chen Z Wickström SL Gao J He X Jing X. Interleukin-33 is a novel immunosuppressor that protects cancer cells from TIL killing by a macrophage-mediated shedding mechanism. Advanced Sci (2021) 8:2101029. doi: 10.1002/advs.202101029 PMC8564439 34486239

[173] Rybakin V Stas M Ugarte-Berzal E Noppen S Vandooren J Van Aelst I. Gelatinase b/matrix metalloproteinase-9 and other neutrophil proteases switch off interleukin-2 activity. Biochem J (2019) 476:2191–208. doi: 10.1042/BCJ20180382 31262730

[174] Xue D Moon B Liao J Guo J Zou Z Han Y. A tumor-specific pro-IL-12 activates preexisting cytotoxic T cells to control established tumors. Sci Immunol (2022) 7:eabi6899. doi: 10.1126/sciimmunol.abi6899 34995098 PMC9009736

[175] Stuelten CH Byfield SD Arany PR Karpova TS Stetler-Stevenson WG Roberts AB. Breast cancer cells induce stromal fibroblasts to express MMP-9 via secretion of TNF-α and TGF-β. J Cell Sci (2005) 118:2143–53. doi: 10.1242/jcs.02334 15855236

[176] Radons J Falk W Schubert T. Interleukin-10 does not affect IL-1-induced interleukin-6 and metalloproteinase production in human chondrosarcoma cells, SW1353. Int J Mol Med (2006) 17:377–83. doi: 10.3892/ijmm.17.2.377 16391840

[177] Albini A Bruno A Noonan DM Mortara L. Contribution to tumor angiogenesis from innate immune cells within the tumor microenvironment: Implications for immunotherapy. Front Immunol (2018) 9:527. doi: 10.3389/fimmu.2018.00527 29675018 PMC5895776

[178] Lugano R Ramachandran M Dimberg A. Tumor angiogenesis: causes, consequences, challenges and opportunities. Cell Mol Life Sci (2020) 77:1745–70. doi: 10.1007/s00018-019-03351-7 PMC7190605 31690961

[179] Viallard C Larrivée B. Tumor angiogenesis and vascular normalization: alternative therapeutic targets. Angiogenesis (2017) 20:409–26. doi: 10.1007/s10456-017-9562-9 28660302

[180] Sun B Zhang D Zhang S Zhang W Guo H Zhao X. Hypoxia influences vasculogenic mimicry channel formation and tumor invasion-related protein expression in melanoma. Cancer Lett (2007) 249:188–97. doi: 10.1016/j.canlet.2006.08.016 16997457

[181] Swayampakula M McDonald PC Vallejo M Coyaud E Chafe SC Westerback A. The interactome of metabolic enzyme carbonic anhydrase IX reveals novel roles in tumor cell migration and invadopodia/MMP14-mediated invasion. Oncogene (2017) 36:6244–61. doi: 10.1038/onc.2017.219 PMC5684442 28692057

[182] Wei X Chen Y Jiang X Peng M Liu Y Mo Y. Mechanisms of vasculogenic mimicry in hypoxic tumor microenvironments. Mol Cancer (2021) 20:7. doi: 10.1186/s12943-020-01288-1 33397409 PMC7784348

[183] Gilkes DM Semenza GL Wirtz D. Hypoxia and the extracellular matrix: drivers of tumour metastasis. Nat Rev Cancer (2014) 14:430–9. doi: 10.1038/nrc3726 PMC4283800 24827502

[184] Scherer RL McIntyre JO Matrisian LM. Imaging matrix metalloproteinases in cancer. Cancer Metastasis Rev (2008) 27:679–90. doi: 10.1007/s10555-008-9152-9 18465089

[185] Zhang X Liu R Yuan Q Gao F Li J Zhang Y. The precise diagnosis of cancer Invasion/Metastasis *via* 2D laser ablation mass mapping of metalloproteinase in primary cancer tissue. ACS Nano (2018) 12:11139–51. doi: 10.1021/acsnano.8b05584 30359513

[186] Sun L Xie S Ji X Zhang J Wang D Lee SJ. MMP-2-responsive fluorescent nanoprobes for enhanced selectivity of tumor cell uptake and imaging. Biomater Sci (2018) 6:2619–26. doi: 10.1039/C8BM00593A 30109310

[187] Cho H-J Lee S Park S-J Lee Y-D Jeong K Park JH. Tumor microenvironment-responsive fluorogenic nanoprobe for ratiometric dual-channel imaging of lymph node metastasis. Colloids Surfaces B: Biointerfaces (2019) 179:9–16. doi: 10.1016/j.colsurfb.2019.03.047 30928802

[188] Zhan Y Ling S Huang H Zhang Y Chen G Huang S. Rapid unperturbed-tissue analysis for intraoperative cancer diagnosis using an enzyme-activated NIR-II nanoprobe. Angew Chem Int Ed (2021) 60:2637–42. doi: 10.1002/anie.202011903 33015947

[189] Coussens LM Fingleton B Matrisian LM. Matrix metalloproteinase inhibitors and cancer–trials and tribulations. Science (2002) 295:2387–92. doi: 10.1126/science.1067100 11923519

[190] Han X Li H Zhou D Chen Z Gu Z. Local and targeted delivery of immune checkpoint blockade therapeutics. Acc Chem Res (2020) 53:2521–33. doi: 10.1021/acs.accounts.0c00339 PMC8177058 33073988

[191] Chen G Chen Z Wen D Wang Z Li H Zeng Y. Transdermal cold atmospheric plasma-mediated immune checkpoint blockade therapy. Proc Natl Acad Sci USA (2020) 117:3687–92. doi: 10.1073/pnas.1917891117 PMC7035610 32029590

[192] Tian T Wang J Yin F Wu M He W Wang X. Activation of cascade-like antitumor immune responses through *In situ* doxorubicin stimulation and blockade of checkpoint coinhibitory receptor TIGIT. Adv Healthcare Materials (2022) 11:2102080. doi: 10.1002/adhm.202102080 34655464

[193] Santos PM Butterfield LH. Dendritic cell–based cancer vaccines. JI (2018) 200:443–9. doi: 10.4049/jimmunol.1701024 PMC5880540 29311386

[194] Rosenberg SA Yang JC Restifo NP. Cancer immunotherapy: moving beyond current vaccines. Nat Med (2004) 10:909–15. doi: 10.1038/nm1100 PMC1435696 15340416

[195] Aspord C Leccia M-T Charles J Plumas J. Plasmacytoid dendritic cells support melanoma progression by promoting Th2 and regulatory immunity through OX40L and ICOSL. Cancer Immunol Res (2013) 1:402–15. doi: 10.1158/2326-6066.CIR-13-0114-T 24778133

[196] Marczynska J Ozga A Wlodarczyk A Majchrzak-Gorecka M Kulig P Banas M. The role of metalloproteinase ADAM17 in regulating ICOS ligand–mediated humoral immune responses. JI (2014) 193:2753–63. doi: 10.4049/jimmunol.1302893 25108021

[197] Lownik JC Luker AJ Damle SR Cooley LF El Sayed R Hutloff A. ADAM10-mediated ICOS ligand shedding on b cells is necessary for proper T cell ICOS regulation and T follicular helper responses. JI (2017) 199:2305–15. doi: 10.4049/jimmunol.1700833 PMC5605448 28814605

[198] Maurer DM Adamik J Santos PM Shi J Shurin MR Kirkwood JM. Dysregulated NF-κB–dependent ICOSL expression in human dendritic cell vaccines impairs T-cell responses in patients with melanoma. Cancer Immunol Res (2020) 8:1554–67. doi: 10.1158/2326-6066.CIR-20-0274 PMC8018573 33051240

[199] Hong M Clubb JD Chen YY. Engineering CAR-T cells for next-generation cancer therapy. Cancer Cell (2020) 38:473–88. doi: 10.1016/j.ccell.2020.07.005 32735779

[200] Sterner RC Sterner RM. CAR-T cell therapy: current limitations and potential strategies. Blood Cancer J (2021) 11:69. doi: 10.1038/s41408-021-00459-7 33824268 PMC8024391

[201] Milone MC Xu J Chen S-J Collins MA Zhou J Powell DJ. Engineering-enhanced CAR T cells for improved cancer therapy. Nat Cancer (2021) 2:780–93. doi: 10.1038/s43018-021-00241-5 PMC8412433 34485921

[202] Wang D Starr R Chang W-C Aguilar B Alizadeh D Wright SL. Chlorotoxin-directed CAR T cells for specific and effective targeting of glioblastoma. Sci Transl Med (2020) 12:eaaw2672. doi: 10.1126/scitranslmed.aaw2672 32132216 PMC7500824

[203] Veiseh M Gabikian P Bahrami S-B Veiseh O Zhang M Hackman RC. Tumor paint: A Chlorotoxin:Cy5.5 bioconjugate for intraoperative visualization of cancer foci. Cancer Res (2007) 67:6882–8. doi: 10.1158/0008-5472.CAN-06-3948 17638899

[204] Deshane J Garner CC Sontheimer H. Chlorotoxin inhibits glioma cell invasion *via* matrix metalloproteinase-2. J Biol Chem (2003) 278:4135–44. doi: 10.1074/jbc.M205662200 12454020

[205] Mardomi A Abediankenari S. Matrix metalloproteinase 8: Could it benefit the CAR-T cell therapy of solid tumors?- a- commentary on therapeutic potential. Cancer Microenviron (2018) 11:93–6. doi: 10.1007/s12307-018-0208-2 PMC6008262 29589335

[206] Santamaria S de Groot R. Monoclonal antibodies against metzincin targets: Metzincins and mAbs. Br J Pharmacol (2019) 176:52–66. doi: 10.1111/bph.14186 29488211 PMC6284333

[207] Fischer T Riedl R. Inhibitory antibodies designed for matrix metalloproteinase modulation. Molecules (2019) 24:2265. doi: 10.3390/molecules24122265 31216704 PMC6631688

[208] Appleby TC Greenstein AE Hung M Liclican A Velasquez M Villaseñor AG. Biochemical characterization and structure determination of a potent, selective antibody inhibitor of human MMP9. J Biol Chem (2017) 292:6810–20. doi: 10.1074/jbc.M116.760579 PMC5399127 28235803

[209] Sun T Zhang YS Pang B Hyun DC Yang M Xia Y. Engineered nanoparticles for drug delivery in cancer therapy. Angew Chem Int Ed (2014). 53(46):12320–64. doi: 10.1002/anie.201403036 25294565

[210] Gonzalez-Avila G Sommer B García-Hernandez AA Ramos C Flores-Soto E. Nanotechnology and matrix metalloproteinases in cancer diagnosis and treatment. Front Mol Biosci (2022) 9:918789. doi: 10.3389/fmolb.2022.918789 35720130 PMC9198274

[211] Han Q-J Lan X-T Wen Y Zhang C-Z Cleary M Sayyed Y. Matrix metalloproteinase-9-Responsive surface charge-reversible nanocarrier to enhance endocytosis as efficient targeted delivery system for cancer diagnosis and therapy. Adv Healthc Mater (2021) 10(9):e2002143. doi: 10.1002/adhm.202002143 33694329

[212] Xia F Niu J Hong Y Li C Cao W Wang L. Matrix metallopeptidase 2 targeted delivery of gold nanostars decorated with IR-780 iodide for dual-modal imaging and enhanced photothermal/photodynamic therapy. Acta Biomaterialia (2019) 89:289–99. doi: 10.1016/j.actbio.2019.03.008 30851455

